# The Eaf3 chromodomain acts as a pH sensor for gene expression by altering its binding affinity for histone methylated-lysine residues

**DOI:** 10.1042/BSR20191958

**Published:** 2020-02-20

**Authors:** Masahiko Okuda, Yoshifumi Nishimura

**Affiliations:** Graduate School of Medical Life Science, Yokohama City University, 1-7-29 Suehiro-cho, Tsurumi-ku, Yokohama 230-0045, Japan

**Keywords:** chromodomain, histidine, histone methylation, intracellular pH, NMR

## Abstract

During gene expression, histone acetylation by histone acetyltransferase (HAT) loosens the chromatin structure around the promoter to allow RNA polymerase II (Pol II) to initiate transcription, while de-acetylation by histone deacetylase (HDAC) tightens the structure in the transcribing region to repress false initiation. Histone acetylation is also regulated by intracellular pH (pH_i_) with global hypoacetylation observed at low pH_i_, and hyperacetylation, causing proliferation, observed at high pH_i_. However, the mechanism underlying the pH_i_-dependent regulation of gene expression remains elusive. Here, we have explored the role of the chromodomain (CD) of budding yeast Eaf3, a common subunit of both HAT and HDAC that is thought to recognize methylated lysine residues on histone H3. We found that Eaf3 CD interacts with histone H3 peptides methylated at Lys4 (H3K4me, a promoter epigenetic marker) and Lys36 (H3K36me, a coding region epigenetic marker), as well as with many dimethyl-lysine peptides and even arginine-asymmetrically dimethylated peptides, but not with unmethylated, phosphorylated or acetylated peptides. The Eaf3 CD structure revealed an unexpected histidine residue in the aromatic cage essential for binding H3K4me and H3K36me. pH titration experiments showed that protonation of the histidine residue around physiological pH controls the charge state of the aromatic cage to regulate binding to H3K4me and H3K36me. Histidine substitution and NMR experiments confirmed the correlation of histidine p*K*_a_ with binding affinity. Collectively, our findings suggest that Eaf3 CD functions as a pH_i_ sensor and a regulator of gene expression via its pH_i_-dependent interaction with methylated nucleosomes.

## Introduction

Post-translational modifications of histones play an essential role in the regulation of transcription, DNA repair, replication and heterochromatin formation by changing the chromatin structure. For example, histones are specifically acetylated and methylated to induce gene expression [1,2]. *Saccharomyces cerevisiae* shows a global pattern of histone H3 and H4 acetylation, whereby acetylation is higher at promoters and lower in coding regions [3,4]. Acetylation loosens the chromatin structure, allowing RNA polymerase II (Pol II) to easily access the promoter; in transcribing regions, by contrast, de-acetylation tightens the chromatin structure, meaning that Pol II can read the gene information but cannot access the gene to start transcription from a cryptic site. Similar to these patterns of acetylation, lysine 4 of histone H3 (H3K4) is methylated by Set1 methyltransferase at promoter sites, whereas Set2 methyltransferase mediates H3K36 methylation in coding regions [5].

In addition to the acetylation pattern for gene expression, histone acetylation is also affected by intracellular pH (pH_i_) in both normal and cancer cells [6]. When pH_i_ decreases, histones are globally de-acetylated by histone deacetylases (HDACs), and the released acetate anions are co-exported with protons from cells by monocarboxylate transporters to maintain pH_i_. Further decreases in pH_i_ have been shown to trigger apoptosis [7]. Alongside the rising pH_i_ that occurs when resting cells are induced to proliferate, differentiate and progress in the cell cycle [8], global acetylation of histone increases.

Although an apparent relation between pH_i_ and histone acetylation responsible for gene expression has been reported, the pH_i_-dependent molecular mechanism of gene expression due to histone acetylation and methylation remains elusive. In terms of the relation between acetylation and methylation, Eaf3 (essential Sas2-related acetyltransferase1-associated factor 3) is a key protein in *S. cerevisiae*. Eaf3 is a component of both the NuA4 histone acetyltransferase (HAT) complex and the Rpd3S HDAC complex. It is thought that the N-terminal chromodomain (CD) of Eaf3 recognizes H3K36 methylation, which then positions Rpd3S on the nucleosome to suppress intragenic transcription initiation [9–11]. However, binding between Eaf3 CD and methylated H3K36 (H3K36me) is relatively weak; therefore, it is thought that Eaf3 CD determines the specificity for H3K36me but the strong binding affinity is cooperatively mediated by the Rco1 subunit of Rpd3S via its plant homeodomain (PHD) [12,13]. Because of the weak interaction, the structure of Eaf3 CD bound to the H3K36me peptide was determined by NMR using a fusion protein of the CD and a model H3K36me peptide [14].

Notably, Eaf3 is also a subunit of the NuA4 HAT complex, which recognizes H3K4me at promoter site nucleosomes. In fact, Eaf3 CD can also bind weakly to H3K4me, facilitating recruitment of NuA4 to the promoter region. Similar to Rpd3S, the PHD of the Yng2 subunit of NuA4 plays an essential cooperative role in binding of Eaf3 CD to H3K4me [15,16]. Thus, Eaf3 CD seems to have no strong sequence specificity and can bind to both H3K4me and H3K36me, while the specific recruitment of NuA4 and Rpd3S to H3K4me and H3K36me, respectively, is cooperatively determined by the PHD of the respective Yng2 and Rco1 subunits. Nevertheless, the cooperative effect of Eaf3 is important because cells lacking Eaf3 have an even distribution of histone acetylation levels across the genome, whereas wild-type (WT) cells have higher histone acetylation levels at promoter regions than at coding sequences [17].

Here, we have explored the role of Eaf3 CD binding to H3K4me and H3K36me. We examined the complexes of Eaf3 CD bound to lysine-methylated histones, which showed that Eaf3 CD is a unique module containing a histidine, His18, in its binding pocket for methylated lysine residues of histones, even though a positively charged residue would be expected to repulse the positively charged histone methyl-lysine. Protonation of His18 was found to critically control both the binding activity and the pH sensitivity of Eaf3 CD: at high pH_i_, the deprotonation of Eaf3 CD causes strong binding to H3K4me at promoters via NuA4, and to H3K36me at transcribing regions via Rpd3S. Thus, the role of Eaf3 CD is to act as a sensor of pH_i_ to control gene expression, rather than to act as a determinant of binding between H3K4me and H3K36me, which is achieved by other components in NuA4 and Rpd3S.

## Materials and methods

### Purification of Eaf3 CD

WT and mutants of Eaf3 CD (residues 1−120) were expressed as hexa-histidine-tagged products in pET15b vectors (Novagen) in *Escherichia coli* BL21(DE3)pLysS (Novagen). Lysed supernatant was loaded onto a Ni-nitrilotriacetic acid (NTA)-agarose (Qiagen) column. Eluted His-tagged Eaf3 CD was then treated with thrombin to remove the 6xHis-tag. The sample was again loaded onto the Ni-NTA agarose column. Fractions passing through the column were concentrated and applied onto Superdex75 (GE Healthcare).

### Chemical shift perturbation

Histone H3 and H4 peptides were synthesized and purchased from Qiagen and SIGMA Genosys. The histone peptide was added to 0.1 mM ^15^N-labeled Eaf3 CD at a molar ratio of 1:4, or 1:8, or 1:16 (Eaf3:histone) in 10 mM potassium phosphate (pH 6.8), 20 mM NaCl, and 5 mM deuterated DTT dissolved in 90% H_2_O/10% D_2_O. ^1^H,^15^N-HSQC spectra were acquired before and after peptide addition at 25°C on a Bruker AVANCE-600 spectrometer equipped with a cryogenic probe. Chemical shift change was calculated as ∆δ = {(∆δ^1^H)^2^ + (∆δ^15^N/5)^2^}^1/2^.

### Peptide titration

For titration, 0.1 mM ^15^N-labeled WT or mutant Eaf3 CD (protein) was titrated with increasing amounts of unlabeled histone H3 peptide, trimethylated lysine, or asymmetrically dimethylated arginine (ligand) in 10 mM potassium phosphate (pH 6.8), 20 mM NaCl, and 5 mM deuterated DTT dissolved in 90% H_2_O/10% D_2_O at 25°C on a Bruker AVANCE-600 spectrometer equipped with a cryogenic probe. In the experiments using H3K36me3 peptide at different pH, the titration was performed in 10 mM sodium acetate at pH 5.2; 10 mM potassium phosphate at pH 6.0 or 7.5; or 10 mM Tris containing 20 mM NaCl at pH 8.5. NMR signal changes were measured by recording ^1^H,^15^N-HSQC spectra before and after each addition. Where an interaction was detected, the ligand was added at a molar ratio of 1:4, 1:8, 1:12, 1:16, 1:20, 1:24, 1:28, 1:40, 1:52, 1:64 and 1:88, where no interaction was observed, ligand was added at 1:8, 1:16, 1:28, 1:40, 1:52, 1:64 and 1:88. Chemical shift change ∆δ was computed by using the formula ∆δ = {(∆δ^1^H)^2^ + (∆δ^15^N/5)^2^}^1/2^ and plotted as a function of molar ratio. *K*_d_ values were calculated by employing the following nonlinear regression fitting function:

$$\Delta \text{δ}=\Delta {\text{δ}}_{\max}({\text{K}}_{\text{d}}+{\left[\text{P}\right]}_{\text{t}}+{\left[\text{L}\right]}_{\text{t}}-{\left\{{\left({\text{K}}_{\text{d}}+{\left[\text{P}\right]}_{\text{t}}+{\left[\text{L}\right]}_{\text{t}}\right)}^{2}-(4{\left[\text{P}\right]}_{\text{t}}{\left[\text{L}\right]}_{\text{t}})\right\}}^{1/2})/2{\left[\text{P}\right]}_{\text{t}}$$

where ∆δ_max_ is the maximal change in chemical shift, and [*P*]_*t*_ and [*L*]_*t*_ are the total concentrations of protein and ligand, respectively.

### NMR structure determination

For the structure determination, 1.0–1.5 mM Eaf3 CD in 10 mM potassium phosphate (pH 6.8), 20 mM NaCl and 5 mM deuterated DTT dissolved in either 90% H_2_O/10% D_2_O or 99.9% D_2_O was used. NMR experiments were performed at 25°C on a Bruker AVANCE-500, AVANCE-600 or AVANCE-800 spectrometer equipped with a cryogenic probe. Backbone and side chain resonances were assigned by using CBCA(CO)NH, CBCANH, HN(CO)CA, HNCA, HN(CA)CO, HNCO, HBHA(CO)NH, HCCH-COSY, HCCH-TOCSY, CCCONH and HCCCONH [18]. Aromatic side chain resonances were assigned by using DQF-COSY, TOCSY, (HB)CB(CGCD)HD, (HB)CB(CGCDCE)HE [19], CG(CB)H, CG(CD)H and CG(CDCE)H [20]. Stereospecific assignments were obtained from a combination of HNHB, HN(CO)HB, HNCG, HN(CO)CG [21] and ^13^C-edited NOESY-HSQC (τ_m_ = 50 ms) [18]. Distance restraints were obtained from NOESY, ^15^N-edited NOESY-HSQC (τ_m_ = 150 ms) and ^13^C-edited NOESY-HSQC (τ_m_ = 50 and 100 ms). Side-chain torsion angles, χ1 and χ2, were obtained from a combination of HNHB, HN(CO)HB, HNCG, HN(CO)CG [21] and ^13^C-edited NOESY-HSQC (τ_m_ = 50 ms) [18]. Hydrogen bond restraints were obtained by backbone amide H/D-exchange experiments. Spectra were processed by NMRPipe [22] and analyzed by NMRView [23].

### Structure calculation

Interproton distance restraints derived from NOE intensities were grouped into four distance ranges, 1.8–2.7 Å (1.8–2.9 Å for NOEs involving HN protons), 1.8–3.3 Å (1.8–3.5 Å for NOEs involving HN protons), 1.8–5.0 Å and 1.8–6.0 Å, corresponding to strong, medium, weak and very weak NOEs, respectively. The upper limit was corrected for constraints involving methyl groups, aromatic ring protons and non-stereospecifically assigned methylene protons. Dihedral angle restraints for φ and ψ were obtained by analyzing the backbone chemical shifts with TALOS [24]. χ1 and χ2 angles were restrained ± 30 ° for three side-chain rotamers. Structure calculations were performed by distance geometry and simulated annealing by using Xplor-NIH [25,26], and all structures were subjected to water refinement [27]. A total of 200 structures were calculated. Structural statistics for the 20 best structures are summarized in Supplementary Table S2. Structures were analyzed and displayed using PROCHECK-NMR [28], MOLMOL [29] and PyMOL (Schroedinger).

### NMR relaxation analysis

The order parameter, *S*^2^, was obtained from the model-free analysis assuming axially symmetric rotation. The backbone ^15^N relaxation parameters of Eaf3 CD were measured by using a ^15^N-labeled sample of 0.30 mM at 25°C on a Bruker AVANCE-600 (^15^N frequency, 60.8 MHz) spectrometer equipped with a cryogenic probe. Relaxation durations of 24, 256, 512, 768, 1024, 1280, 1536, 2048 and 2560 ms for the longitudinal relaxation rates (*R*1), and 16.98, 33.95, 50.93, 67.90, 101.86, 135.81, 203.71 and 339.52 ms for the transverse relaxation rates (*R*2) were used. Heteronuclear ^15^N-{^1^H} NOE experiments were recorded in the presence and absence of proton saturation, which was achieved with a 5.0 s duration consisting of 120° ^1^H pulses applied every 5.0 ms. *R*1 and *R*2 were obtained by fitting peak intensities at a series of relaxation durations to an exponential decay curve using CurveFit [30]. The uncertainties of the peak intensities were estimated by using duplicated data from the shortest relaxation delay. The uncertainties of the relaxation rates were determined by CurveFit using a Monte–Carlo simulation. The steady-state ^15^N-{^1^H} NOE values were determined from peak intensity ratios obtained from spectra acquired with and without proton saturation. The uncertainties were determined from the standard deviation in background noise levels by using NMRView [23]. Initial values of the overall correlation time and the axially symmetric rotational diffusion tensor were estimated by using r2r1_diffusion [31]. Model-free analysis was performed by using Modelfree [30] and FAST ModelFree [32].

### pH titration

A series of ^1^H,^15^N-HSQC spectra for 0.1–1.1 mM ^15^N-labeled Eaf3 CD were recorded at different pH ranging from 5.2 to 10.5 at 25°C on a Bruker AVANCE-600 spectrometer equipped with a cryogenic probe. The observed chemical shift (δ_obs_) was plotted as a function of pH. p*K*_a_ values were calculated by a nonlinear least-squares fit of the pH titration curves using the following equation,

$${\text{δ}}_{\text{obs}}=\frac{\left[{\text{δ}}_{\text{l}}+{\text{δ}}_{\text{h}}\times 10^{\left(\text{pH-pKa}\right)}\right]}{\left[1+10^{\left(\text{pH-pKa}\right)}\right]},$$

where δ_l_ and δ_h_ represent the chemical shift values at extreme low and high pH, respectively.

### PRE

A 4-fold molar excess of the spin label reagent MTSL (1-oxy-2,2,5,5-tetramethyl-D3-pyrroline-3-methyl) methanethiosulfonate (Toronto Research Chemicals) dissolved in acetonitrile was added to H3K36me3 peptide (residues 32-41) with a Gly33Cys substitution (SIGMA Genosys), dissolved in buffer [10 mM potassium phosphate (pH 6.8), 20 mM NaCl], and incubated at 25°C for 16 h in the dark. Unreacted MTSL was removed by using a PD MidiTrap G-10 column (GE Healthcare) equilibrated with distilled water. The spin labeled peptide eluted with water was lyophilized and dissolved in buffer, and the pH was adjusted to 6.8. A paramagnetic sample was obtained by adding MTSL-H3K36me3 peptide to ^15^N-labeled Eaf3 CD dissolved in buffer at a molar ratio of 1:1 (final concentration 0.3 mM), and the ^1^H,^15^N-HSQC spectrum was recorded. A diamagnetic sample was generated by reducing the paramagnetic sample with a 3.2-fold molar excess of ascorbate (Wako) at 25°C for 4 h in the dark. After the reduction, the ^1^H,^15^N-HSQC spectrum was recorded.

## Results

### Methyl-specific binding of Eaf3 CD

**Figure 1:**
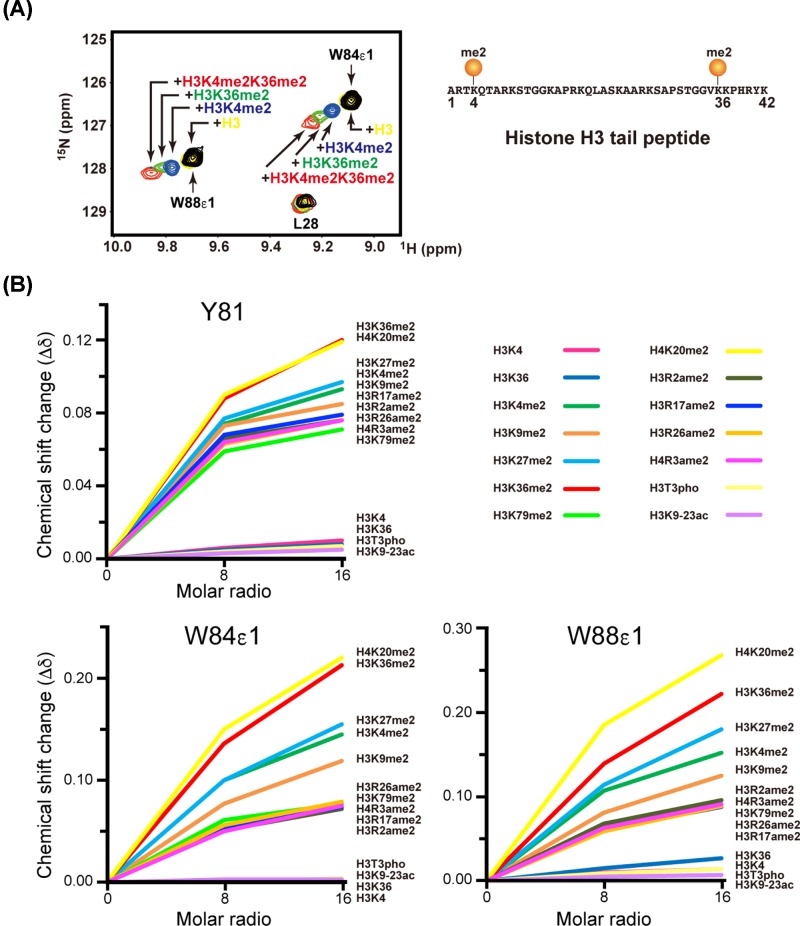
Perturbation of the NMR chemical shift of Eaf3 CD by various post-translationally modified histone peptides (**A**) Overlays of an expanded region of the ^1^H,^15^N-HSQC spectrum of Eaf3 CD before (black) and after the addition of a 4-fold excess of histone H3 peptide (residues 1-42): H3 (yellow), H3K4me2 (blue), H3K36me2 (green) and H3K4me2K36me2 (red). me2: di-methylated. (**B**) Chemical shift change (∆δ) of the backbone amide group of Tyr81 and side-chain imino groups of Trp84 and Trp88 at a molar ratio of 1:8 and 1:16. The added histone peptides are shown on the right. ame2: asymmetrically di-methylated, pho: phosphorylated, ac: acetylated.

It has been reported that Eaf3 CD binds weakly to the H3K4me peptide as well as the H3K36me peptide [9,10,14,33]. To confirm this, we monitored NMR signal changes of ^15^N-labeled Eaf3 CD upon the addition of histone peptides by using ^1^H,^15^N HSQC spectroscopy. Initially, we examined the methylation-dependent specificity of binding to the N-terminal fragment (residues 1−42) of histone H3 (H3) (Figure 1A and Supplementary Table S1). The addition of a 4-fold excess of an H3 peptide di-methylated (me2) at K4, K36, or both lysine residues gave rise to a small but significant signal change of specific residues such as Tyr81, Trp84 and Trp88, which formed a binding pocket (Supplementary Figure S1a). By contrast, the addition of a 4-fold excess of unmodified H3 peptide did not result in any altered signals.

To characterize the weak binding of Eaf3 CD in more detail, we observed the interaction of Eaf3 CD with various histone peptides (Figure 1B and Supplementary Table S1). Eaf3 CD interacted with many dimethyl-lysine peptides, and even with arginine-asymmetrically dimethylated (ame2) peptides, but not with unmethylated, phosphorylated (pho) or acetylated (ac) peptides. Thus, the binding of Eaf3 CD to histone is highly methylation-specific but promiscuous.

### Eaf3 CD binds preferentially to trimethylated peptides and to H3K36me2/3

**Figure 2:**
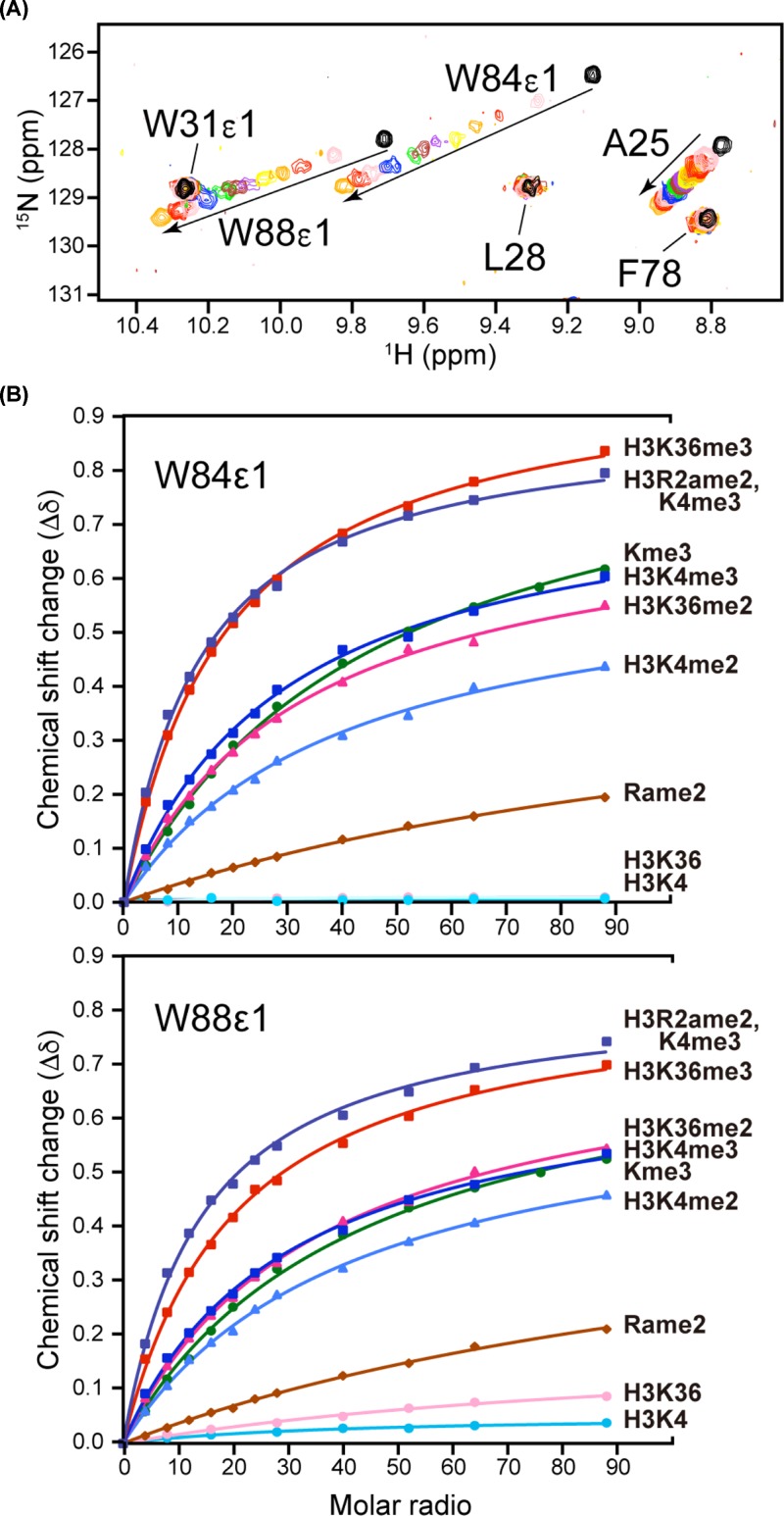
NMR titration of Eaf3 CD with K4- or K36-methylated histone H3 peptide (**A**) Overlays of an expanded region of the ^1^H,^15^N-HSQC spectrum of Eaf3 CD titrated with H3K36me3 peptide. (**B**) Titration curves for the side chain imino groups from Trp84 and Trp88 of Eaf3 CD. The added histone peptides and amino acids are shown on the right. Kme3: tri-methyl-lysine, Rame2: asymmetrically di-methylated arginine.

Because the NuA4 and Rpd3S complexes are closely related to H3K4me and H3K36me, respectively, we focused on these methylation marks and evaluated their quantitative binding to Eaf3 CD by NMR titration (Table 1 and Figure 2). We found that Eaf3 CD bound more strongly (i) to the trimethylated form than to the dimethylated form of both H3K4 and H3K36 and (ii) to di- and trimethylated H3K36 than to the corresponding methylated forms of H3K4. In addition, binding to H3K4me3 was strengthened by R2ame2 modification, but weakened by T3pho modification. Although the different binding features observed were interesting, in all cases the dissociation constant (*K*_d_) values were in the millimolar range. Thus, Eaf3 CD can be categorized as a very weak histone methyl-lysine-binding module.

**Table 1:** Dissociation constant (*K*_d_) for binding of Eaf3 CD to histone H3 peptide

Histone	*K*_d_ (μM)
Kme3*	4440 ± 230
H3K36	N.B.
H3K36me2	3350 ± 200
H3K36me3	1880 ± 130
H3K4	N.B.
H3K4me2	4040 ± 110
H3K4me3	2900 ± 10
H3R2ame2K4me3	1360 ± 20
H3T3phoK3me3	6520 ± 480
Rame2*	>10,000

* amino acid, N.B.: not bound.

### A unique histidine residue in the binding site

All methylated-lysine histone-binding modules have a characteristic binding cage consisting of two to four aromatic residues (Phe, Tyr and Trp), which contact a methylammonium moiety through cation–π and van der Waals interactions [34].

The structure of Eaf3 CD has been previously determined and the binding of Eaf3 to H3K36me found to be weak [14,33]. We re-examined both the dynamic and static structures of Eaf3 CD by using NMR spectroscopy (Supplementary Table S2 and Figure S1a). The present NMR structure was consistent with the previous solved structures, although it resembled the crystal structure more than the solution one (Supplementary Figure S2). Unlike HP1 and Polycomb CDs, where a methylated H3 tail is inserted in a cleft, the corresponding cleft in Eaf3 CD is occupied by β strand S1, and helix H2 is positioned above the S1 strand (Supplementary Figure S1c). In addition, relaxation analysis indicated that there is restricted backbone motion on the picosecond to nanosecond timescale for all regions except for the N- and C-termini (residues 1−8 and 115−120) and a long loop between β strands S3 and S4 (residues 43−57) (Supplementary Figure S1d). The two turns between β strands S1 and S2 and between S5 and S6, which form the binding pocket (Supplementary Figure S1a, marked by dashed line), show a relatively low degree of flexibility as normal (Supplementary Figure S1d, marked by dashed lines). It is therefore clear that the origin of the weak binding of Eaf3 CD is not related to the dynamic character of this domain.

**Figure 3:**
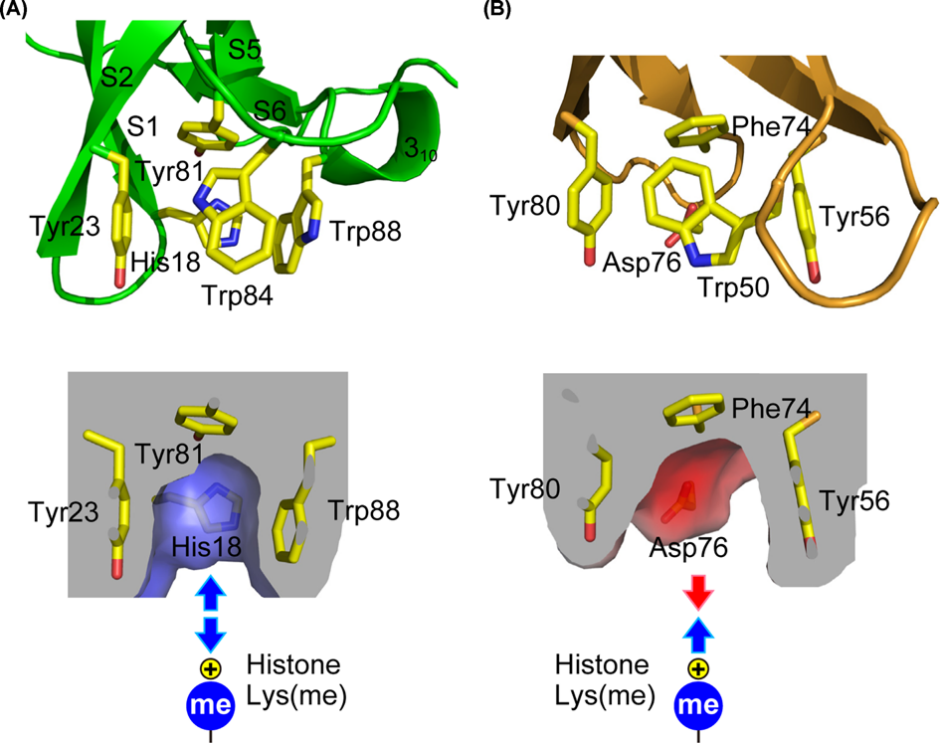
Structural comparison of aromatic binding sites for methyl-lysine of the histone tail (**A**) The aromatic binding cage of Eaf3 CD. (**B**) The aromatic binding cage of the Phf19 Tudor domain [35]. Top: residues that form the binding cage. Bottom: sliced view of the molecular surface showing electrostatic potential (blue: positive, red: negative).

Eaf3 CD possesses an aromatic cage formed by Tyr81 at the bottom with Tyr23, Trp84 and Trp88 around the walls (Figure 3A). A previous study of Eaf3 CD bound to a H3K36me2 analog, in which Eaf3 CD was engineered to link to a H3K36 fragment to a chemically incorporated methyl-lysine analogue produced by cysteine alkylation, revealed that these four aromatic residues (Tyr23, Tyr81, Trp84 and Trp88) surround the dimethyl-lysine analog [14]. In fact, each of the four aromatic residues was found to be essential for the interaction because the alanine mutants Y23A, Y81A, W84A and W88A, each of which maintain the proper conformation (Supplementary Figure S3), failed to bind to a trimethylated (me3) H3K36, H3K4me3 or H3R2ame2K4me3 peptide (Table 2 and Supplementary Figure S4). By contrast, W84Y and two alanine mutants of Gly19 and Gln82, both of which are near to the aromatic cage but not involved in direct binding, maintained the binding ability of Eaf3 CD.

**Table 2:** Dissociation constant (*K*_d_) for the binding of Eaf3 CD variants to methylated histone H3 peptide

Eaf3 CD	H3K36me3	H3K4me3	H3R2ame2K4me3
	*K*_d_ (μM)	*K*_d_ (μM)	*K*_d_ (μM)
Wild-type	1880 ± 130	2900 ± 10	1360 ± 20
G19A	760 ± 40	800 ± 70	430 ± 40
Y23A	N.B.	N.B.	N.B.
Y81A	N.B.	N.B.	N.B.
Q82A	1650 ± 70	2240 ± 280	1260 ± 60
W84A	N.B.	N.B.	N.B.
W84Y	1200 ± 60	1590 ± 280	760 ± 20
W88A	N.B.	N.B.	N.B.

N.B.: not bound.

Taken together, the above results indicate that Eaf3 CD *per se* has few direct contacts with histone residues and relies heavily on the interaction with a methyl-lysine. We therefore focused on the residues inside the binding cage of Eaf3 CD. To identify unique features, we first compared the structures of histone methyl-lysine peptide complexes determined so far. Supplementary Table S3 summarizes the results of a systematic survey of residues in which the heavy atoms of the side-chains of the methyl-lysine-binding domains are within 6 Å of the Nζ atom of the methyl-lysine of histone. As expected, all histone-binding domains contain aromatic residues [34]. Curiously, however, Eaf3 CD possesses a histidine, His18, and not an acidic residue as the charged amino acid (Figure 3A and Supplementary Table S4). In general, a histidine residue will be protonated at physiological pH, which would produce a repulsive Coulombic barrier to interacting with the positively charged methyl-lysine of histone. Contrary to Eaf3 CD, acidic residues are often observed in the binding aromatic cages of other methyl-lysine-binding modules (Supplementary Table S3 and Figure 3B). Notably, the electrostatically unfavorable histidine residue in the binding cage is not limited to yeast Eaf3, and is seen in other species such as animals and plants (Supplementary Figure S5).

### Physiological p*K*_a_ of His18 in the binding site of Eaf3 CD

**Figure 4:**
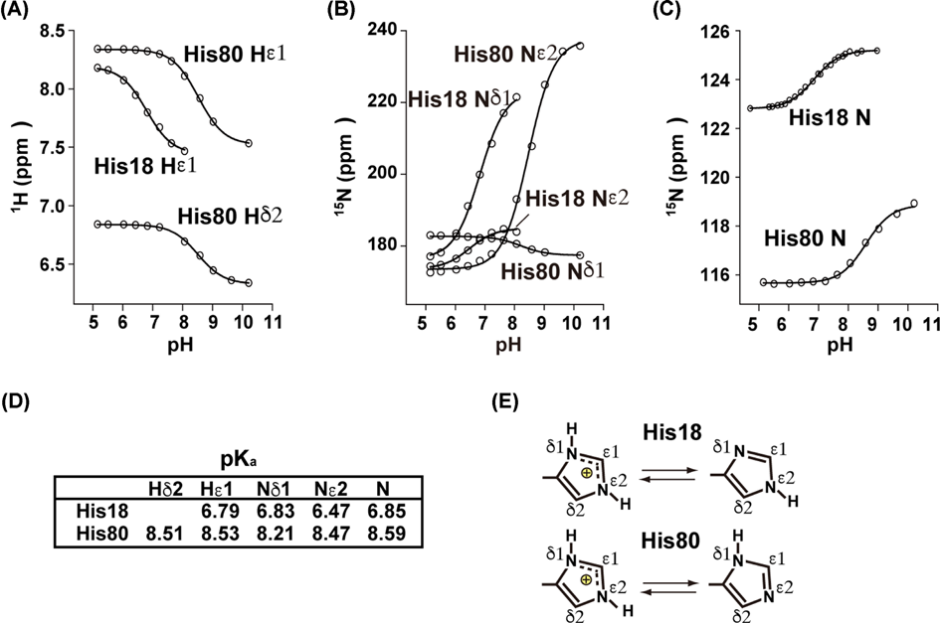
p*K*_a_ values and tautomeric states of the histidine residues of Eaf3 CD Shown are the pH titration curves for ^1^H and ^15^N atoms of the two histidine residues of Eaf3 CD. (**A**) Side chain Hδ2 and Hε1. (**B**) Side-chain Nδ1 and Nε2. (**C**) Main-chain N. (**D**) Calculated p*K*_a_ values. (**E**) Determined tautomeric states of the two histidine residues.

The structural analysis suggested that the unusual His18 residue in the aromatic cage might be a key determinant for the weak interactions of Eaf3 CD. However, a histidine residue has a wide range of acid dissociation constant (p*K*_a_) values depending on its environment. Next, therefore, we determined the p*K*_a_ value of His18 (Figure 4A–D) and its tautomeric state (Figure 4E) by using NMR [36]. We confirmed that no disruption of structure was caused by the pH changes throughout the experiments (not shown). The observed p*K*_a_ value of His18 was approximately 6.8 (Figure 4D), indicating that the binding activity of the aromatic cage of Eaf3 CD is sensitive to changes in pH under physiological conditions.

### Protonation state of His18 facilitates pH-dependent binding of Eaf3 CD

**Figure 5:**
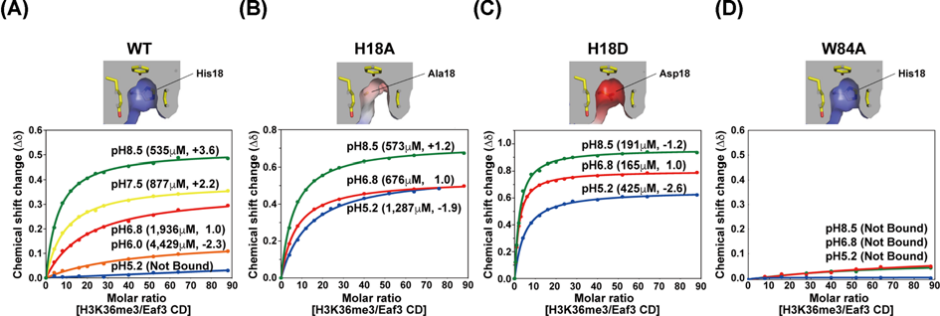
NMR titration of Eaf3 CD with H3K36me3 peptide at different pH (**A**) WT Eaf3 CD, (**B**) H18A Eaf3 CD, (**C**) H18D Eaf3 CD and (**D**) W84A Eaf3 CD. Titration curves are plotted for Ala25 of Eaf3 CD. The calculated *K*_d_ and fold changes for binding at pH 6.8 are given in parentheses.

To verify the pH-dependent binding of Eaf3 CD suggested by the above observations, we examined the interaction of Eaf3 CD with methylated histone peptide under different pH (Figure 5A). An increase in pH from 6.8 to 7.5 enhanced the binding of Eaf3 CD to H3K36me3 2.2-fold. Upon a further increase to pH 8.5, the binding was enhanced a further 3.6-fold. In contrast, a decrease from pH 6.8 to 6.0 weakened the binding 2.3-fold. Decreasing the pH to 5.2, where almost all Eaf3 CD molecules would contain protonated His18, abolished the binding. Thus, the protonation state of His18 is negatively correlated with the binding activity of Eaf3 CD. At a physiological pH of 6.8, almost half of all Eaf3 CD molecules will be protonated at His18 and thus in an inactive binding form, while the rest will be deprotonated at His18 and therefore in an active binding form.

We further confirmed the necessity of protonation of His18 in the observed pH sensitivity of binding by examining the binding activity of H18A and H18D at pH 6.8, 8.5 and 5.2 (Figure 5B,C). In contrast with the alanine mutants of the four cage aromatic residues, none of which showed binding (Table 2), H18A showed 2.9-fold stronger binding to the H3K36me3 peptide as compared with the WT peptide at pH 6.8 (Figure 5A,B). Relative to pH 6.8, the binding of H18A to H3K36me3 peptide was slightly enhanced by a factor of 1.2-fold at pH 8.5, and decreased by 1.9-fold at pH 5.2 (Figure 5B), although pH 5.2 did not result in a complete loss of activity of Eaf3 CD, unlike WT (Figure 5A). H18D enhanced the binding to H3K36me3 peptide even more (Figure 5C,B), with an 11.7-fold increase relative to WT at pH 6.8 (Figure 5A,C). H18D maintained high-binding activity at pH 8.5 and to a lesser extent at pH 5.2 (Figure 5C). In addition, the electrostatic state in the aromatic binding cage of the mutant proteins altered the preference for H3K36me3 (Supplementary Figure S6). As described above (Table 1), WT Eaf3 CD showed a preference for H3K36me3 over H3K36me2 (Supplementary Figure S6a). H18A exhibited the same preference (Supplementary Figure S6b), whereas H18D showed a preference for the dimethyl form over the trimethyl one (Supplementary Figure S6c). For binding to H3K36me2 peptide, H18D exhibited a 71.1-fold stronger binding as compared with WT (Supplementary Figure S6c and a). Thus, the introduction of negative charge into the binding cage of Eaf3 CD greatly augmented the binding activity, disrupted the pH sensitivity and altered the preference to H3K36me3.

We also tested the pH dependence of W84A as a representative of alanine mutants of the four binding aromatic residues in the binding site. Similar to the observations at pH 6.8 (Table 2), W84A showed no binding at pH 8.5 or at pH 5.2, demonstrating the pH independence of mutant W84A regardless of His18 de-protonation (Figure 5D).

### Dual function of Eaf3 CD as a pH_i_ sensor and a histone-binding module

The above findings showed that His18 is the crucial structure determinant of the weak binding of Eaf3 CD. At the same time, they demonstrated a novel function of Eaf3 CD as a pH_i_ sensor with histone methyl-lysine-binding ability. We therefore investigated this dual function from the viewpoint of the structure by using paramagnetic relaxation enhancement (PRE) experiments. In PRE, effects are observed depending only on the distance between the paramagnetic center and the proton; thus, it is possible to obtain structural information about low-population states in an exchanging system such as that of Eaf3 CD and H3K36me3 peptide [37].

**Figure 6:**
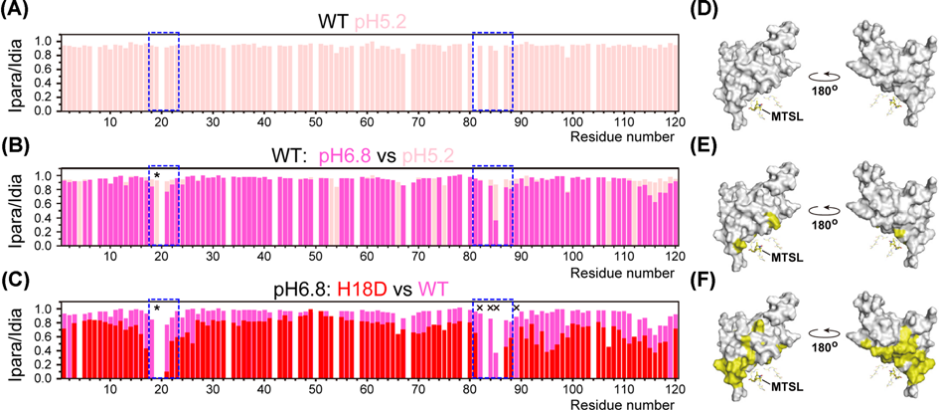
Paramagnetic relaxation enhancement profiles of Eaf3 CD mixed with MTSL spin-labeled H3K36me3 peptide (**A**–**C**) Intensity ratio (*I*_para_/*I*_dia_) of ^15^N-labeled Eaf3 CD residues when mixed with H3K36me3 peptide labeled with either paramagnetic (para) or diamagnetic (dia) MTSL. Th intensity ratio is normalized with the maximum at 1.0. Blue dotted line indicates the position of the aromatic binding cage. (**A**) WT Eaf3 CD, measured at pH 5.2 (pink). (**B**) Comparison between WT Eaf3 CD at pH 6.8 (magenta) and WT Eaf3 CD at pH 5.2 (pink). (**C**) Comparison between H18D Eaf3 CD at pH 6.8 (red) and WT Eaf3 CD at pH 6.8 (magenta). Asterisks indicate residues absent in the paramagnetic state. ‘X’ indicates a residue absent in both paramagnetic and diamagnetic states. Blank spaces are either proline residues or residues for which peak intensity was not measured because the signals overlapped or were unassigned. (**D**–**F**) Residues exhibiting *I*_para_/*I*_dia_ <0.7 (yellow) mapped on the structure. (**D**) WT Eaf3 CD, measured at pH 5.2. (**E**) WT Eaf3 CD, measured at pH 6.8. (**F**) H18D Eaf3 CD, measured at pH 6.8. The position of MTSL is determined by back calculation using the PRE data of H18D [37].

The addition of an H3K36me3 peptide carrying an MTSL spin label to 0.3 mM ^15^N-labeled Eaf3 CD at a molar ratio of 1:1 gave rise to only subtle chemical shift changes of the backbone amide signals for a few residues of Eaf3 CD (Figure 6). At pH 5.2, no PRE effects were observed, indicating no binding (Figure 6A,D). At pH 6.8, by contrast, signals from several specific residues around the binding site were broadened upon addition of the paramagnetically labeled H3K36me3 peptide (Figure 6B,E). For the H18D mutation at pH 6.8, many broadened signals were also observed in the binding site and surrounding area (Figure 6C,F). Taking all the findings together, we conclude that Eaf3 CD is a previously unknown pH_i_ sensor that detects a change in pH_i_ and simultaneously converts it into methylated histone-binding ability via protonation of His18 in the binding site.

## Discussion

### Eaf3 CD offers pH sensitivity to the regulation of HAT/HDAC activity

**Figure 7:**
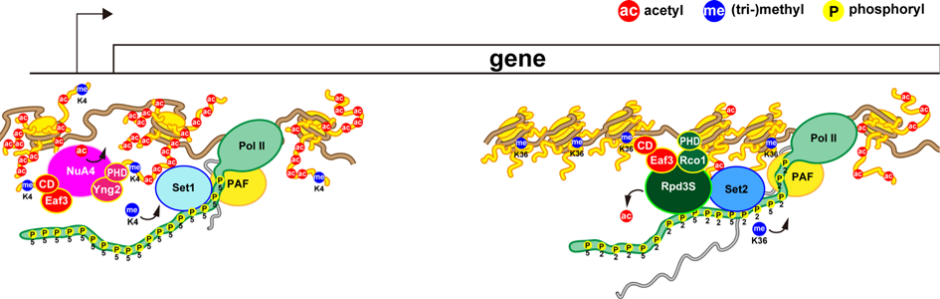
Schematic view of the regulation of gene expression in yeast cells At promoter regions, Set1 methyltransferase (COMPASS complex), which is associated with the Ser5-phosphorylated C-terminal domain (CTD) of the largest subunit of the Pol II and PAF transcription elongation complex, methylates H3K4 of a nucleosome; NuA4 histone acetyltransferase is then recruited to the H3K4me-nucleosome and activated by the cooperative binding of Eaf3 CD, Yng2 PHD, and probably specific domains of the other subunits of NuA4; finally, NuA4 acetylates the nucleosome for efficient transcription initiation. In coding regions, Set2 methyltransferase, which is associated with the Ser2,Ser5-phosphorylated CTD of Pol II, methylates H3K36 of a nucleosome; Rpd3S histone deacetylase is then recruited to the H3K36me-nuclesome through its association with the Set2 and Ser2,Ser5-phosphorylated CTD of Pol II, and activated by cooperative binding of Eaf3 CD and Rco1 PHD; finally, Rpd3S deacetylates the nucleosome to repress spurious transcription from cryptic start sites. To reduce complexity, many of the other chromatin and transcription factors are not depicted.

As described above and in previous studies [9,10,14,33], Eaf3 CD itself has high methylation specificity but low sequence specificity; therefore, combinatorial actions with the PHD of Yng2 in NuA4 and with the PHD of Rco1 in Rpd3S are necessary for the overall affinity and specificity of the H3K4me- and H3K36me-nucleosome interaction [12,16], and activation [15,38,39] of the complexes (Figure 7). A biochemical study previously demonstrated that Rpd3S preferentially binds di-nucleosomes by contacting two nucleosomes simultaneously via Eaf3 CD and Rco1 PHD [40]. Deuterium exchange mass spectrometry study also found that Rpd3S undergoes conformational changes upon contact with nucleosomes, and the Sin3-interacting domain of Rco1 allosterically stimulates preferential binding of Eaf3 CD to H3K36me [13]. These findings strongly suggest that the precise positioning of the complex on nucleosomes by multiple domains is crucial for regulation of the enzymatic activity. Our findings now open up the possibility that Eaf3 CD provides pH sensitivity to this regulation. We suspect that Eaf3 CD is needed to swiftly respond to cellular signals of pH_i_ perturbation and coordinate co-transcriptional chromatin remodeling. For instance, during cell proliferation when the pH_i_ increases, subsets of genes should be activated, requiring both dynamic opening of promoter regions by HAT for easy access of Pol II, and tight closing of coding regions by HDAC after Pol II passes through to avoid aberrant gene transcripts. Via His18, Eaf3 CD senses increasing pH_i_ and simultaneously strengthens its affinity for methylated nucleosomes, thereby placing NuA4 and Rpd3S in the appropriate position on the nucleosomes in combination with other subunits. Activated NuA4 on promoter regions and Rpd3S on coding regions establish an environment for efficient transcription. The generation of acetate during the histone deacetylation process might promote dissociation of Rpd3S from nucleosomes in the gene bodies. Further studies are needed to verify this notion.

### Generality of the function of the pH_i_ sensor activity of Eaf3 CD beyond yeast cells

In addition to Eaf3, *S. cerevisiae* has two well-known CD-containing proteins: Chd1, an ATP-dependent chromatin remodeling factor; and Esa1, the catalytic subunit of HAT NuA4. In previous studies, we found that both CDs are structurally incompetent to bind to lysine-methylated histone [41,42]. Given the fact that Eaf3 is a limited CD protein possessing methylated histone-binding ability in yeast, its pH-responsive interactions may have a fundamental role. This idea is supported by the fact that the significance of fluctuations in pH_i_ is not limited to yeast. In higher eukaryotes, changes in pH_i_ have been correlated with cell proliferation [43], differentiation [44], cell cycle progression [45], apoptosis [7] and cancer [46]. In this regard, it is worth noting that His18 of Eaf3 CD is highly conserved (Supplementary Figure S5) and the corresponding histidine of Eaf3 orthologs – namely, His21 in human MRG15, His62 in *Arabidopsis* MRG2, and His37 in rice MRG701 – actually lies at a structurally equivalent position [47–49]. In addition, the interactions between MRG15 CD and methyl-lysine peptides have been found to be strongly sensitive to the pH used for NMR titrations, with little or no interaction between MRG15 CD and H3K36me3 peptide at acidic pH [50]. On the basis of these observations, we predict that all MRG protein CDs essentially functions as a pH_i_ sensor identical with the role of Eaf3 CD described here.

Finally, to explore pH_i_ sensor activity and the correlation between pH_i_ and histone acetylation, we focused here on the histone methyl-lysine-binding domain and one of its histidine residues. However, this does not mean that Eaf3 CD is the only domain that acts as a pH_i_ sensor. It is noteworthy that the PHDs of human Death Inducer Obliterator 3 (Dido3) and its *Drosophila* ortholog PPS use a histidine to bind trimethylated H3K4 and exhibit pH dependence [51,52]. The *Arabidopsis* SHH1 tandem Tudor domain also contains a histidine in its aromatic binding cage for methylated H3K9 [53]. We speculate that other chromatin-associated proteins might use a similar pH-responsive mechanism to regulate chromatin structure.

## Supplementary Material

Supplementary Figures S1-S6 and Tables S1-S4

## Abbreviations

ac: acetylated
ame2: arginine-asymmetrically dimethylated
CD: chromodomain
Eaf3: essential Sas2-related acetyltransferase1-associated factor 3
H3: histone H3
H4: histone H4
HAT: histone acetyl transferase
HDAC: histone deacetylase
HSQC: heteronuclear single quantum coherence
*K*_d_: dissociation constant
me2: dimethylated
me3: trimethylated
NMR: nuclear magnetic resonance
NOE: nuclear overhauser effect
pH_i_: intracellular pH
pho: phosphorylated
Pol II: RNA polymerase II
p*K*_a_: acid dissociation constant
PRE: paramagnetic relaxation enhancement
WT: wild-type

## References

[1] Kouzarides T. (2007) Chromatin modifications and their function. Cell 128, 693–705 10.1016/j.cell.2007.02.005 17320507

[2] Strahl B.D. and Allis C.D. (2000) The language of covalent histone modifications. Nature 403, 41–45 10.1038/47412 10638745

[3] Pokholok D.K., Harbison C.T., Levine S., Cole M., Hannett N.M., Lee T.I. et al. (2005) Genome-wide map of nucleosome acetylation and methylation in yeast. Cell 122, 517–527 10.1016/j.cell.2005.06.026 16122420

[4] Robyr D., Suka Y., Xenarios I., Kurdistani S.K., Wang A., Suka N. et al. (2002) Microarray deacetylation maps determine genome-wide functions for yeast histone deacetylases. Cell 109, 437–446 10.1016/S0092-8674(02)00746-8 12086601

[5] Hampsey M. and Reinberg D. (2003) Tails of intrigue: phosphorylation of RNA polymerase II mediates histone methylation. Cell 113, 429–432 10.1016/S0092-8674(03)00360-X 12757703

[6] McBrian M.A., Behbahan I.S., Ferrari R., Su T., Huang T.W., Li K. et al. (2013) Histone acetylation regulates intracellular pH. Mol. Cell 49, 310–321 10.1016/j.molcel.2012.10.025 23201122 PMC3893119

[7] Lagadic-Gossmann D., Huc L. and Lecureur V. (2004) Alterations of intracellular pH homeostasis in apoptosis: origins and roles. Cell Death Differ. 11, 953–961 10.1038/sj.cdd.4401466 15195071

[8] Grinstein S., Rotin D. and Mason M.J. (1989) Na+/H+ exchange and growth factor-induced cytosolic pH changes. Role in cellular proliferation. Biochim. Biophys. Acta 988, 73–97 10.1016/0304-4157(89)90004-X 2535787

[9] Carrozza M.J., Li B., Florens L., Suganuma T., Swanson S.K., Lee K.K. et al. (2005) Histone H3 methylation by Set2 directs deacetylation of coding regions by Rpd3S to suppress spurious intragenic transcription. Cell 123, 581–592 10.1016/j.cell.2005.10.023 16286007

[10] Joshi A.A. and Struhl K. (2005) Eaf3 chromodomain interaction with methylated H3-K36 links histone deacetylation to Pol II elongation. Mol. Cell 20, 971–978 10.1016/j.molcel.2005.11.021 16364921

[11] Keogh M.C., Kurdistani S.K., Morris S.A., Ahn S.H., Podolny V., Collins S.R. et al. (2005) Cotranscriptional set2 methylation of histone H3 lysine 36 recruits a repressive Rpd3 complex. Cell 123, 593–605 10.1016/j.cell.2005.10.025 16286008

[12] Li B., Gogol M., Carey M., Lee D., Seidel C. and Workman J.L. (2007) Combined action of PHD and chromo domains directs the Rpd3S HDAC to transcribed chromatin. Science 316, 1050–1054 10.1126/science.1139004 17510366

[13] Ruan C., Lee C.H., Cui H., Li S. and Li B. (2015) Nucleosome contact triggers conformational changes of Rpd3S driving high-affinity H3K36me nucleosome engagement. Cell Rep. 10, 204–215 10.1016/j.celrep.2014.12.027 25578729 PMC4359074

[14] Xu C., Cui G., Botuyan M.V. and Mer G. (2008) Structural basis for the recognition of methylated histone H3K36 by the Eaf3 subunit of histone deacetylase complex Rpd3S. Structure 16, 1740–1750 10.1016/j.str.2008.08.008 18818090 PMC2582589

[15] Steunou A.L., Cramet M., Rossetto D., Aristizabal M.J., Lacoste N., Drouin S. et al. (2016) Combined action of histone reader modules regulates NuA4 local acetyltransferase function but not its recruitment on the genome. Mol. Cell. Biol. 36, 2768–2781 10.1128/MCB.00112-16 27550811 PMC5086519

[16] Su W.P., Hsu S.H., Chia L.C., Lin J.Y., Chang S.B., Jiang Z.D. et al. (2016) Combined Interactions of plant homeodomain and chromodomain regulate NuA4 activity at DNA double-strand breaks. Genetics 202, 77–92 10.1534/genetics.115.184432 26564157 PMC4701104

[17] Reid J.L., Moqtaderi Z. and Struhl K. (2004) Eaf3 regulates the global pattern of histone acetylation in *Saccharomyces cerevisiae*. Mol. Cell. Biol. 24, 757–764 10.1128/MCB.24.2.757-764.2004 14701747 PMC343795

[18] Cavanagh J., Fairbrother W.J., Palmer A.G. III and Skelton N.J. (2006) Protein NMR Spectroscopy: Principles and Practice, 2nd edn, Academic Press, San Diego, CA

[19] Yamazaki T., Forman-Kay J.D. and Kay L.E. (1993) Two-dimensional NMR experiments for correlating ^13^Cβ and ^1^Hδ/ε chemical shifts of aromatic residues in ^13^C-labeled proteins via scalar coupling. J. Am. Chem. Soc. 115, 11054–11055 10.1021/ja00076a099

[20] Prompers J.J., Groenewegen A., Hilbers C.W. and Pepermans H.A.M. (1998) Two-dimensional NMR experiments for the assignment of aromatic side chains in ^13^C-labaled proteins. J. Magn. Reson. 130, 68–75 10.1006/jmre.1997.1277 9469899

[21] Krichna N.R. and Berliner L.J. (1998) Biological Magnetic Resonance 16, Kluwer Academic/Plenum Publishers, New York

[22] Delaglio F., Grzesiek S., Vuister G.W., Zhu G., Pfeifer J. and Bax A. (1995) NMRPipe: A multidimensional spectral processing system based on UNIX pipes. J. Biomol. NMR 6, 277–293 8520220 10.1007/BF00197809

[23] Johnson B.A. and Blevins R.A. (1994) NMRView: a computer program for the visualization and analysis of NMR data. J. Biomol. NMR 4, 603–614 10.1007/BF00404272 22911360

[24] Cornilescu G., Delaglio F. and Bax A. (1999) Protein backbone angle restraints from searching a database for chemical shift and sequence homology. J. Biomol. NMR 13, 289–302 10.1023/A:1008392405740 10212987

[25] Brünger A.T. (1993) X-PLOR Version 3.1: A System for X-Ray Crystallography and NMR, Yale University Press, New Haven, CT

[26] Schwieters C.D., Kuszewski J.J., Tjandra N. and Clore G.M. (2003) The Xplor-NIH NMR molecular structure determination package. J. Magn. Reson. 160, 65–73 10.1016/S1090-7807(02)00014-9 12565051

[27] Linge J.P., Williams M.A., Spronk C.A., Bonvin A.M. and Nilges M. (2003) Refinement of protein structures in explicit solvent. Proteins 50, 496–506 10.1002/prot.10299 12557191

[28] Laskowski R.A., Rullmann J.A.C., MacArthur M.W., Kaptein R. and Thornton J.M. (1996) AQUA and PROCHECK-NMR: Programs for checking the quality of protein structures solved by NMR. J. Biomol. NMR 8, 477–486 9008363 10.1007/BF00228148

[29] Koradi R., Billeter M. and Wüthrich K. (1996) MOLMOL: A program for display and analysis of macromolecular structures. J. Mol. Graph. 14, 51–55 10.1016/0263-7855(96)00009-4 8744573

[30] Mandel A.M., Akke M. and Palmer A.G. III (1995) Backbone dynamics of *Escherichia coli* ribonuclease HI: correlations with structure and function in an active enzyme. J. Mol. Biol. 246, 144–163 10.1006/jmbi.1994.0073 7531772

[31] Tjandra N., Feller S.E., Pastor R.W. and Bax A. (1995) Rotational diffusion anisotropy of human ubiquitin from ^15^N NMR relaxation. J. Am. Chem. Soc. 117, 12562–12566 10.1021/ja00155a020

[32] Cole R. and Loria J.P. (2003) FAST-Modelfree: a program for rapid automated analysis of solution NMR spin relaxation data. J. Biomol. NMR 26, 203–213 10.1023/A:1023808801134 12766418

[33] Sun B., Hong J., Zhang P., Dong X., Shen X., Lin D. et al. (2008) Molecular basis of the interaction of *Saccharomyces cerevisiae* Eaf3 chromo domain with methylated H3K36. J. Biol. Chem. 283, 36504–36512 10.1074/jbc.M806564200 18984594 PMC2662307

[34] Taverna S.D., Li H., Ruthenburg A.J., Allis C.D. and Patel D.J. (2007) How chromatin-binding modules interpret histone modifications: lessons from professional pocket pickers. Nat. Struct. Mol. Biol. 14, 1025–1040 10.1038/nsmb1338 17984965 PMC4691843

[35] Ballaré C., Lange M., Lapinaite A., Martin G.M., Morey L., Pascual G. et al. (2012) Phf19 links methylated Lys36 of histone H3 to regulation of Polycomb activity. Nat. Struct. Mol. Biol. 19, 1257–1265 10.1038/nsmb.2434 23104054 PMC3926938

[36] Pelton J.G., Torchia D.A., Meadow N.D. and Roseman S. (1993) Tautomeric states of the active-site histidines of phosphorylated and unphosphorylated IIIGlc, a signal-transducing protein from *Escherichia coli*, using two-dimensional heteronuclear NMR techniques. Protein Sci. 2, 543–558 8518729 10.1002/pro.5560020406 PMC2142369

[37] Iwahara J. and Clore G.M. (2006) Detecting transient intermediates in macromolecular binding by paramagnetic NMR. Nature 440, 1227–1230 10.1038/nature04673 16642002

[38] Drouin S., Laramée L., Jacques PÉ., Forest A., Bergeron M. and Robert F. (2010) DSIF and RNA polymerase II CTD phosphorylation coordinate the recruitment of Rpd3S to actively transcribed genes. PLoS Genet. 6, e1001173 10.1371/journal.pgen.1001173 21060864 PMC2965751

[39] Govind C.K., Qiu H., Ginsburg D.S., Ruan C., Hofmeyer K., Hu C. et al. (2010) Phosphorylated Pol II CTD recruits multiple HDACs, including Rpd3C(S), for methylation-dependent deacetylation of ORF nucleosomes. Mol. Cell 39, 234–246 10.1016/j.molcel.2010.07.003 20670892 PMC2937259

[40] Huh J.W., Wu J., Lee C.H., Yun M., Gilada D., Brautigam C.A. et al. (2012) Multivalent di-nucleosome recognition enables the Rpd3S histone deacetylase complex to tolerate decreased H3K36 methylation levels. EMBO J. 31, 3564–3574 10.1038/emboj.2012.221 22863776 PMC3433781

[41] Okuda M., Horikoshi M. and Nishimura Y. (2007) Structural polymorphism of chromodomains in Chd1. J. Mol. Biol. 365, 1047–1062 10.1016/j.jmb.2006.10.039 17098252

[42] Shimojo H., Sano N., Moriwaki Y., Okuda M., Horikoshi M. and Nishimura Y. (2008) Novel structural and functional mode of a knot essential for RNA binding activity of the Esa1 presumed chromodomain. J. Mol. Biol. 378, 987–1001 10.1016/j.jmb.2008.03.021 18407291

[43] Denker S.P., Huang D.C., Orlowski J., Furthmayr H. and Barber D.L. (2000) Direct binding of the Na-H exchanger NHE1 to ERM proteins regulates the cortical cytoskeleton and cell shape independently of H+ translocation. Mol. Cell 6, 1425–1436 10.1016/S1097-2765(00)00139-8 11163215

[44] Boussouf A. and Gaillard S. (2000) Intracellular pH changes during oligodendrocyte differentiation in primary culture. J. Neurosci. Res. 59, 731–739 10.1002/(SICI)1097-4547(20000315)59:6<731::AID-JNR5>3.0.CO;2-G 10700010

[45] Putney L.K. and Barber D.L. (2003) Na-H exchange-dependent increase in intracellular pH times G2/M entry and transition. J. Biol. Chem. 278, 44645–44649 10.1074/jbc.M308099200 12947095

[46] Stock C. and Schwab A. (2009). Protons make tumor cells like clockwork. Pflugers Arch. 458, 981–992 10.1007/s00424-009-0677-8 19437033

[47] Zhang P., Du J., Sun B., Dong X., Xu G., Zhou J. et al. (2006) Structure of human MRG15 chromo domain and its binding to Lys36-methylated histone H3. Nucleic Acids Res. 34, 6621–6628 10.1093/nar/gkl989 17135209 PMC1747190

[48] Bu Z., Yu Y., Li Z., Liu Y., Jiang W., Huang Y. et al. (2014) Regulation of *arabidopsis* flowering by the histone mark readers MRG1/2 via interaction with CONSTANS to modulate FT expression. PLoS Genet. 10, e1004617 10.1371/journal.pgen.1004617 25211338 PMC4161306

[49] Liu Y., Wu H., Yu Y. and Huang Y. (2016) Structural studies on MRG701 chromodomain reveal a novel dimerization interface of MRG proteins in green plants. Protein Cell 7, 792–803 10.1007/s13238-016-0310-5 27638467 PMC5084153

[50] Kumar G.S., Chang W., Xie T., Patel A., Zhang Y., Wang G.G. et al. (2012) Sequence requirements for combinatorial recognition of histone H3 by the MRG15 and Pf1 subunits of the Rpd3S/Sin3S corepressor complex. J. Mol. Biol. 422, 519–531 10.1016/j.jmb.2012.06.013 22728643 PMC3428507

[51] Gatchalian J., Fütterer A., Rothbart S.B., Tong Q., Rincon-Arano H., Sánchez de Diego A. et al. (2013) Dido3 PHD modulates cell differentiation and division. Cell Rep. 4, 148–158 10.1016/j.celrep.2013.06.014 23831028 PMC3967714

[52] Tencer A.H., Gatchalian J., Klein B.J., Khan A., Zhang Y., Strahl B.D. et al. (2017) A unique pH-dependent recognition of methylated histone H3K4 by PPS and DIDO. Structure 25, 1530–1539 10.1016/j.str.2017.08.009 28919441 PMC5679713

[53] Law J.A., Du J., Hale C.J., Feng S., Krajewski K., Palanca A.M. et al. (2013) Polymerase IV occupancy at RNA-directed DNA methylation sites requires SHH1. Nature 498, 385–389 10.1038/nature12178 23636332 PMC4119789

