# Supplementary material for: The Eaf3 chromodomain acts as a pH sensor for gene expression by altering its binding affinity for histone methylated-lysine residues
Source: Biosci Rep. 2020 Feb 20;40(2):BSR20191958. doi: 10.1042/BSR20191958 (PMC7033311; doi:10.1042/BSR20191958)
Supplement: Supplementary Figures S1-S6 and Tables S1-S4 [file BSR-2019-1958_supp.pdf]

Supplementary Figures and Tables

The Eaf3 Chromodomain Acts as a pH Sensor for Gene Expression by Altering its Binding Affinity for Histone Methylated-Lysine Residues

Masahiko Okuda and Yoshifumi Nishimura

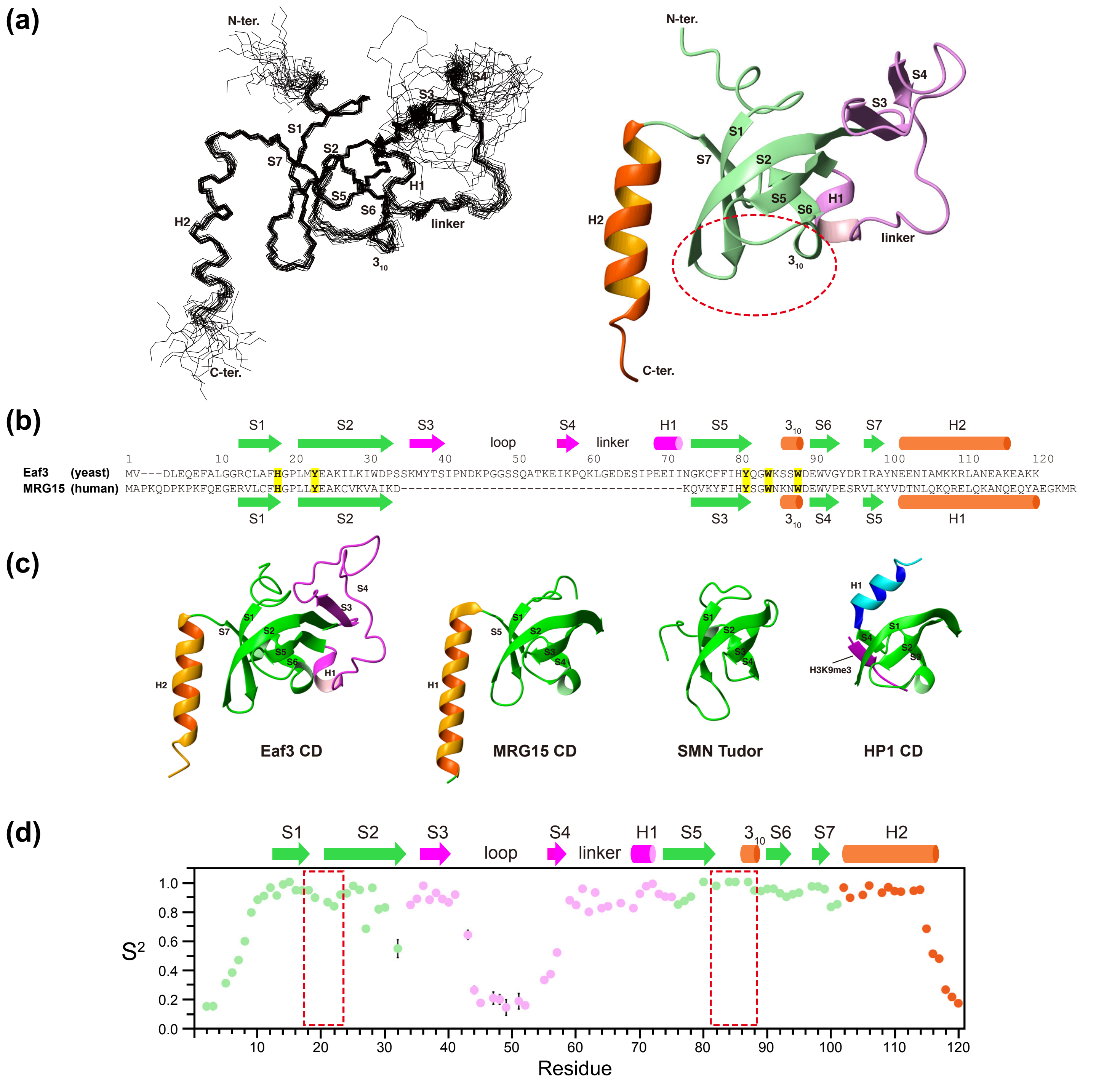

Fig. S1. Structure of Eaf3 CD.

(a) Solution structure of Eaf3 CD. Left: superposition of the ensemble of the 20 lowest-energy structures. Right: ribbon representation of the lowest-energy structure. Red dotted line indicates the position of the aromatic binding cage.

(b) Sequence alignment of the CD of budding yeast Eaf3 and human MRG15. Residues involved in the cage formation are shaded in yellow.

(c) Structural comparison. Eaf3 CD (Protein Data Bank (PDB) ID: 6K5W), MRG15 CD (PDB ID: 2F5K), SMN Tudor (PDB ID: 1G5V), and HP1 CD (PDB ID: 1KNE).

(d) Order parameter S^2 of the backbone of Eaf3 CD. Red dotted line indicates the position of the aromatic binding cage.

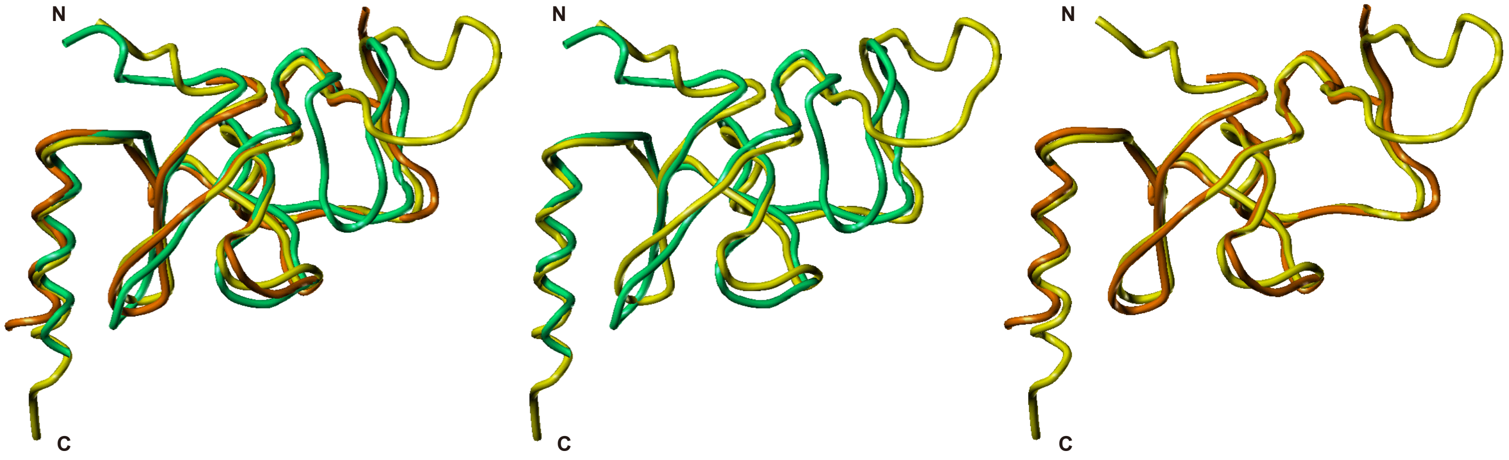

Fig. S2. Structure comparison of Eaf3 CDs.
Structural superposition of Eaf3 CDs. Yellow; this study, PDB ID: 6K5W. Green; PDB ID: 2K3X. Orange; PDB ID: 3K9F. Left; 6K5W v.s. 2K3X v.s. 3K9F. Middle; 6K5W v.s. 2K3X. Right; 6K5W v.s. 3K9F. Structures are superimposed for residues 11–42 and 56–113. R.s.m. deviations for the backbone structure between 6K5W and 2K3X, and between 6K5W and 3K9F were 1.62 Å and 1.07 Å, respectively.

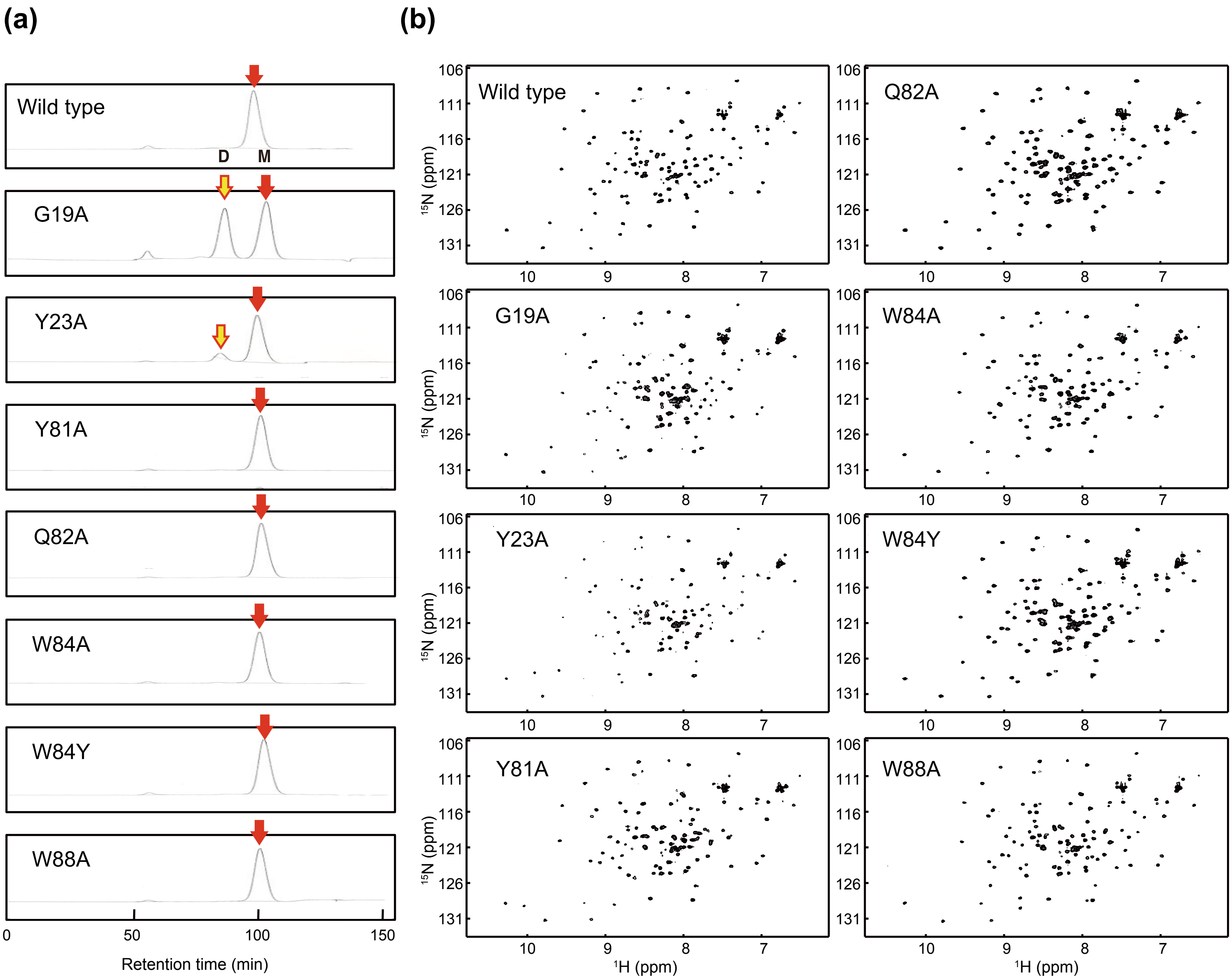

Fig. S3. Preparation of site-directed mutants of Eaf3 CD.
(a) Size exclusion chromatograms of wild-type and mutant Eaf3 CD. D: dimer, M: monomer.
(b) NMR ^{1}H,^{15}N HSQC spectra of wild-type and mutant Eaf3 CD.

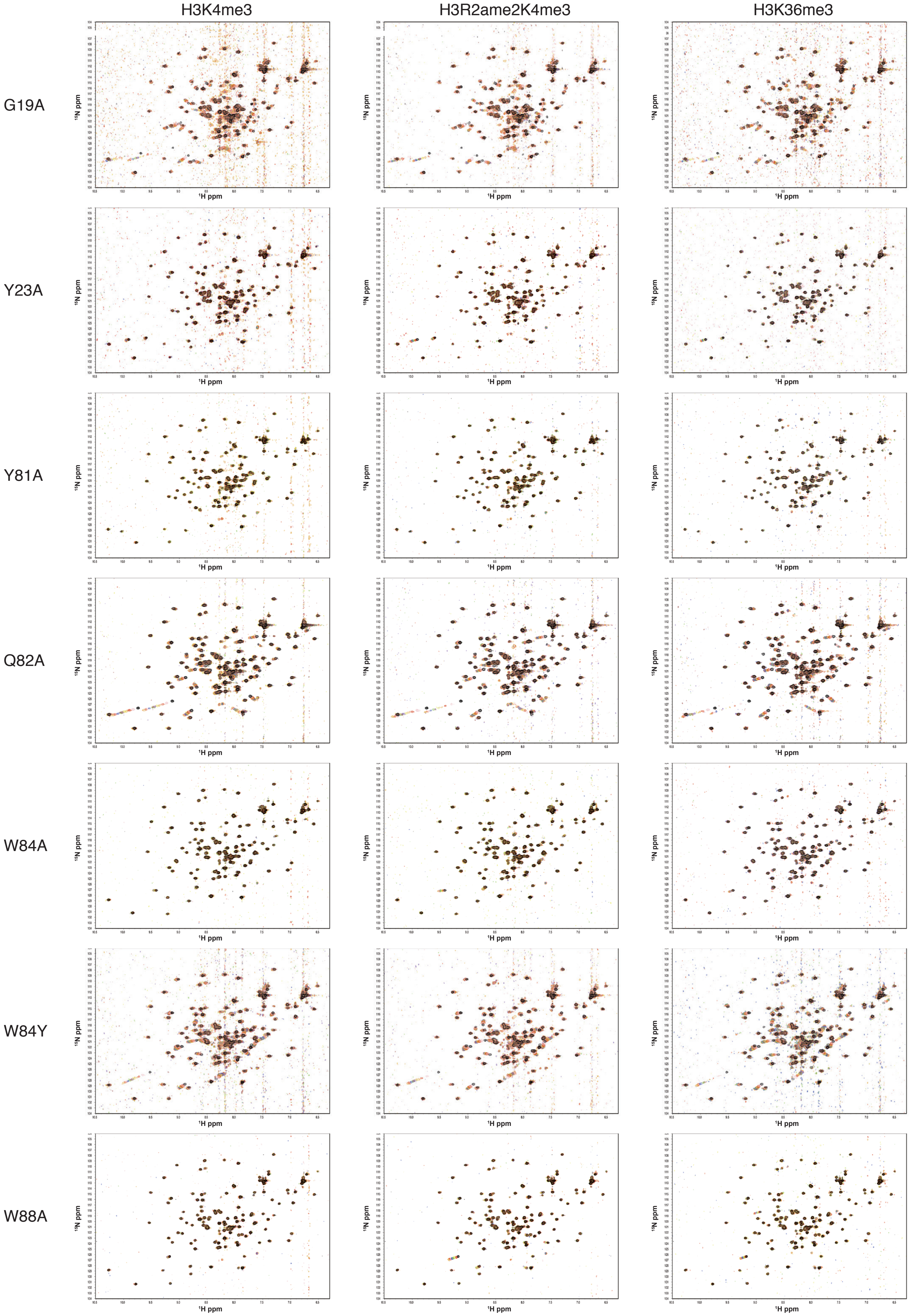

Fig. S4. NMR titration experiments.
Overlays of the ^{1}H,^{15}N-HSQC spectra of Eaf3 CD mutants titrated with methylated H3 peptide. The methylated H3 peptides were added to each Eaf3 CD mutant at up to 88-fold molar excess.

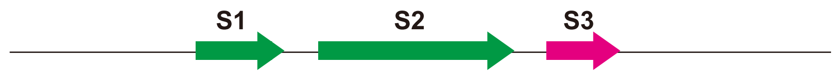


```
                                   1         10        20        30        40        50
Saccharomyces cerevisiae      --------------MVDLEQEFALGGRCLAFHGPLMYEAKILKIWDPSSKMYTSIPNDKPGGSSQATK
Schizosaccharomyces pombe   1 -----------------MAVSYKVNERVLCFHGPLLYEAKIVDTEMKG-------------------- 31
Drosophila melanogaster     1 MGEVKPAKVENYSTGTDANTLFVDGERVLCFHGPLIYEAKVLKTKPDA-------------------- 48
Xenopus laevis              1 -----------MAPKQDPRPKFQDGERVLCFHGPLLYEAKCVKVSIKD-------------------- 37
Bos taurus                  1 -----------MAPKQDPKPKFQEGERVLCFHGPLLYEAKCVKVAIKD-------------------- 37
Rattus norvegicus           1 -----------MAPKQDPKPKFQEGERVLCFHGPLLYEAKCVKVAIKD-------------------- 37
Mus musculus                1 -----------MAPKQDPKPKFQEGERVLCFHGPLLYEAKCVKVAIKD-------------------- 37
Homo sapiens                1 -----------MAPKQDPKPKFQEGERVLCFHGPLLYEAKCVKVAIKD-------------------- 37
Arabidopsis thaliana       31 TNTDCDGEDLPLLLLPAPPGHFEEGERVLAKHSDCFYEAKVLKVEFKD-------------------- 78
Caenorhabditis elegans      1 ---------------MSSKKNFEVGENVACIYKGKPYDAKITDIKTNS-------------------- 33
```

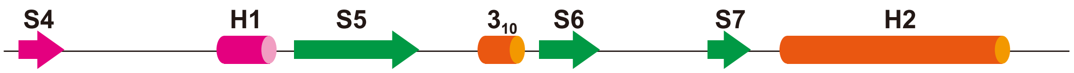


```
                                  60        70        80        90            100       110       120
Saccharomyces cerevisiae    EIKPQKLGEDESIPEEIINGKCFFIHYQGWKSSWDEWVGY----DRIRAYNEENIAMKKRLANEAKEAKK
Schizosaccharomyces pombe   ------------------DVTTYLIHYKGWKNSWDEWVEQ----DRILQWTEENLKTQKELKNAAISTRQ 79
Drosophila melanogaster     ------------------TPVEYYIHYAGWSKNWDEWVPE----NRVLKYNDDNVKRRQELARQCGERSK 96
Xenopus laevis              ------------------KQVKYFIHYSGWNKNWDEWVPE----SRVLKYVDTNLQKQKELQKANQDHYV 85
Bos taurus                  ------------------KQVKYSIHYSGWNKNWDEWVPE----SRVLKYVDTNLQKQRELQKANQKTKK 85
Rattus norvegicus           ------------------KQVKYFIHYSGWNKNWDEWVPE----SRVLKYVDANLQKQRELQKANQEQYA 85
Mus musculus                ------------------KQVKYFIHYSGWNKNWDEWVPE----SRVLKYVDTNLQKQRELQKANQEQYA 85
Homo sapiens                ------------------KQVKYFIHYSGWNKNWDEWVPE----SRVLKYVDTNLQKQRELQKANQEQYA 85
Arabidopsis thaliana        ------------------NEWKYFVHYIGWNKSWDEWIRL----DCLLKHSDENIEKQKEQGLKQQGIKS 126
Caenorhabditis elegans      -----------------DGKELYCVHFKGWNNRYDEKIPVGEEKDRIFKGTASEYAEKHNAELPTTALKP 86
```

Fig. S5. Sequence alignment of the CD of budding yeast Eaf3 and various homologues.
Residues involved in the cage formation are shaded in yellow. The secondary structure of budding yeast Eaf3 CD is shown above the sequence.

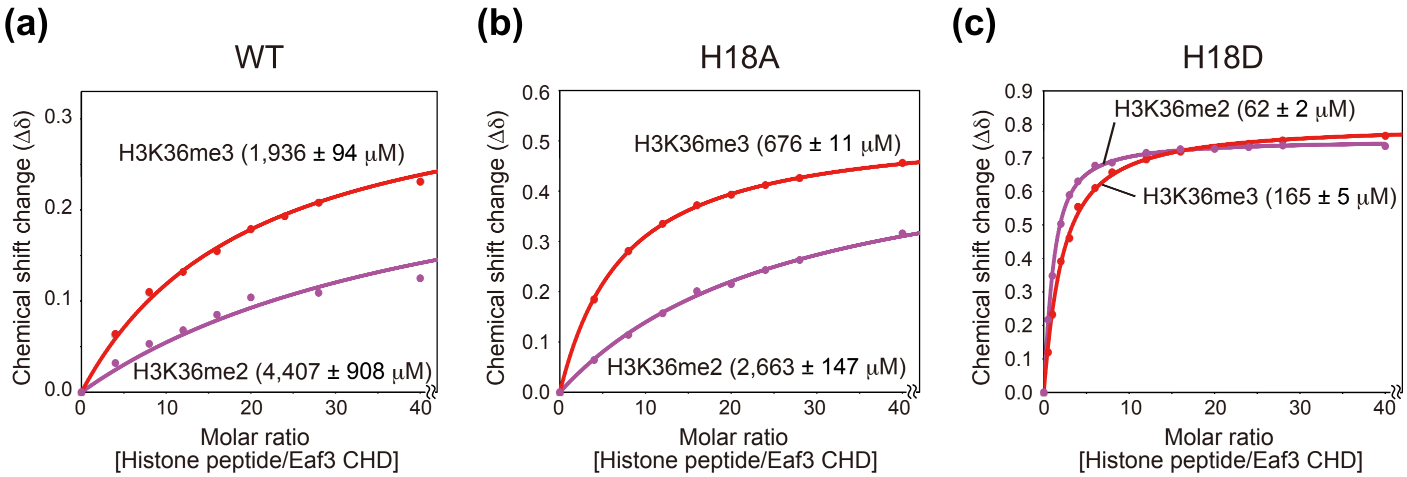

Fig. S6. NMR titration experiment.
Shown are the titration curves for ^{15}N-labeled Eaf3 CD with H3K36me2 and H3K36me3 peptides at pH 6.8. (a) Wild-type Eaf3 CD; (b) H18A mutant; (c) H18D mutant. The titration curves are plotted for Ala25 of Eaf3 CD. Calculated K_d values are given in parentheses.

Table S1. Histone peptides used in the NMR binding experiments

Name	Sequence
NMR chemical shift perturbation experiment	
H3	ARTKQTARKSTGGKAPRKQLASKAARKSAPSTGGVKKPHRYK
H3K4me2	ARTK(me2)QTARKSTGGKAPRKQLASKAARKSAPSTGGVKKPHRYK
H3K36me2	ARTKQTARKSTGGKAPRKQLASKAARKSAPSTGGVK(me2)KPHRYK
H3K4me2K36me2	ARTK(me2)QTARKSTGGKAPRKQLASKAARKSAPSTGGVK(me2)KPHRYK
H3K4	ARTKQTARKSTGGKAPRY
H3K4me2	ARTK(me2)QTARKSTGGKAPRY
H3K9me2	ARTKQTARK(me2)STGGKAPRY
H3K27me2	KQLASKAARK(me2)SAPSTGGVY
H3K36	SAPSTGGVKKPHRYKPGT
H3K36me2	SAPSTGGVK(me2)KPHRYKPGT
H3K79me2	VREIAQDFK(me2)TDLRFQSSY
H4K20me2	KGGAKRHRK(me2)ILRDNIQGY
H3R2ame2	AR(ame2)TKQTARKSTGGKAPRKQLAY
H3R17ame2	ARKSTGGKAPR(ame2)KQLASKAARKY
H3R26ame2	PRKQLASKAAR(ame2)KSAPSTGGVKY
H4R3ame2	SGR(ame2)GKGGKGLGKGGAKRHRKY
H3T3pho	ART(pho)KQTARKSTGGKAPRY
H3K9−23ac	ARK(ac)STGGK(ac)APRK(ac)QLATK(ac)AAY
NMR titration experiment	
H3K4	ARTKQTARKSTGGKAPRY
H3K4me2	ARTK(me2)QTARKSTGGKAPRY
H3K4me3	ARTK(me3)QTARKSTGGKAPRY
H3K36	SAPSTGGVKKPHRYKPGT
H3K36me2	SAPSTGGVK(me2)KPHRYKPGT
H3K36me3	SAPSTGGVK(me3)KPHRYKPGT
H3R2(ame2)K4me3	AR(ame2)TK(me3)QTARKSTGGKAPRY
H3T3(pho)K4me3	ART(ph)K(me3)QTARKSTGGKAPRY

me2: di-methylated, me3: tri-methylated, ame2: asymmetrically di-methylated,
pho: phosphorylated, ac: acetylated.

Table S2. Structural statistics for the 20 best structures of Eaf3 CD

Experimental restraints	
Total NOEs	2480
Intraresidue	110
Sequential (i-j =1)	659
Medium-range (1<i-j<5)	546
Intramolecular long-range (i-j≥5)	1165
Hydrogen bond	23 × 2
Number of dihedral restraints	
φ	59
ψ	58
χ1	23
χ2	3
Statistics for structure calculations	
R.m.s.d. from experimental restraints*	
Distance (Å)	0.038 ± 0.001
Dihedral (°)	0.566 ± 0.078
R.m.s.d. from idealized covalent geometry	
Bonds (Å)	0.0060 ± 0.0001
Angles (°)	0.70 ± 0.02
Improper (°)	0.71 ± 0.03
Coordinate precision (residues 7−41, 56−115)	
Average pairwise r.m.s.d. from the mean structure	
Backbone atoms (Å)	0.64 ± 0.12
Heavy atoms (Å)	1.29 ± 0.17
Ramachandran plot statistics (residues 7−41, 56−115)	
Residues in most favored regions (%)	73.8
Residues in additional allowed regions (%)	23.8
Residues in generously allowed regions (%)	2.4
Residues in disallowed regions (%)	0.0

* None of the structures exhibited distance violations of >0.5 Å or dihedral angle violations of >4°.

Table S3. Residues forming the binding site for methyl-lysine residues in the histone tail

Domain	Protein	Histone	PDB ID	Residues having side-chains within 6 Å of Nζ of methylated lysine		
				Aromatic	Charged	Others
CD	scEaf3	H3K36me2	2K3Y (NMR)	Tyr23 Tyr81 Trp84 Trp88	His18	
	aMRG2	H3K4me3	4PL6 (X-ray)	Tyr67 Tyr87 Trp90 Trp94	His62	
	aMRG2	H3K36me3	4PLI (X-ray)	Tyr67 Tyr87 Trp90 Trp94	His62	
	hMSL3	H4K20me1	3OA6 (X-ray)	Tyr31 Phe56 Trp59 Trp63	Glu21	Pro22
	mHP1	H3K9me2	1GUW (NMR)	Tyr21 Trp42 Phe45	Asp49 Glu53	Thr51
	dHP1	H3K9me2	1KNA (X-ray)	Tyr24 Trp45 Tyr48	Glu52	
	dPC	H3K27me3	1PDQ (X-ray)	Tyr26 Trp47 Trp50 Tyr54		Thr56
	dPC	H3K27me3	1PFB (X-ray)	Tyr26 Trp47 Trp50		Thr56
	spChp1	H3K9me3	2RSN (NMR)	Tyr22 Trp44 Tyr47	Asp51	
	spChp1	H3K9me3	3G7L (X-ray)	Tyr22 Trp44 Tyr47	Asp51	
	hCBX1	H3K9me3	6D07 (X-ray)	Tyr3 Trp24 Phe27	Asp31	Thr33
	hCBX2	H3K27me3	3H91 (X-ray)	Phe12 Trp33 Trp36	His40	Ser42
	hCBX3	H3K9me3	2L11 (NMR)	Phe30 Trp51 Phe54	Asp58 Glu62	Thr60
	hCBX3	H1K26me2	3TZD (X-ray)	Phe26 Phe30 Trp51 Phe54	Asp58	
	hCBX5	H3K9me3	3FDT (X-ray)	Tyr20 Trp41 Phe44	His48	Thr50
	hCBX6	H3K9me3	3GV6 (X-ray)	Phe11 Trp32 Trp35 Tyr39		Thr41
	hCBX6	H3K27me3	3GV6 (X-ray)	Phe11 Trp32 Trp35 Tyr39		Thr41
	hCBX7	H3K9me3	2L12 (NMR)	Phe11 Trp32 Trp35		Gln9 Thr41
	hCBX7	H3K27me3	2L1B (NMR)	Phe11 Trp32 Trp35 Tyr39		Gln9 Thr41
	mCBX7	H3K27me2	2KVM (NMR)	Phe11 Trp32 Trp35 Tyr39		Thr41
	mCBX7	H3K27me3	4X3K (X-ray)	Phe11 Trp32 Trp35 Tyr39		Thr41
	hCBX8	H3K9me3	3I91 (X-ray)	Phe11 Trp32 Trp35 Tyr39		Thr41
	hCHD1	H3K4me3	2B2W (X-ray)	Trp322 Trp325	Glu272	Thr331
	hMPP8	H3K9me3	3QO2 (X-ray)	Phe59 Trp80 Tyr83	Asp87	
	hMPP8	H3K9me3	3R93 (X-ray)	Phe59 Trp80 Tyr83	Asp87	
	maZMET2	H3K9me2	4FT2 (X-ray)	Phe441 Trp466 Tyr469	Glu473	Thr475
	dRHINO	H3K9me3	4QUF (X-ray)	Tyr24 Trp45 Phe48		Asn52 Thr54
	dRHINO	H3K9me3	4U68 (X-ray)	Tyr24 Trp45 Phe48		Asn52 Thr54
	hCDYL2	H1K26me2	5JJZ (X-ray)	Tyr7 Trp29 Tyr32		Thr38
PHD	hBPTF	H3K4me3	2FUU (NMR)	Tyr2869 Tyr2876 Tyr2882 Trp2891		
	hING1	H3K4me3	2QIC (X-ray)	Tyr355 Trp378		Ser362
	mING2	H3K4me3	2G6Q (X-ray)	Tyr215 Trp238		Ser222 Met226
	hING4	H3K4me3	2PNX (X-ray)	Tyr198 Trp221		Ser205 Met209
	hING4	H3K4me3	2VNF (X-ray)	Tyr198 Trp221		Ser205 Met209
	hING5	H3K4me3	3C6W (X-ray)	Tyr188 Trp211		Ser195 Met199
	scYng1	H3K4me3	2JMJ (NMR)	Tyr157 Trp180		Ser164 Met168
	hTAF3	H3K4me3	5WXH (X-ray)	Trp861 Trp866 Trp889	Asp875	Met880
	mTAF3	H3K4me3	2K17 (NMR)	Trp868 Trp891	Asp877	Met882
	mRAG2	H3K4me3	2V89 (X-ray)	Tyr415 Trp453		Gly414 Met443
	hPYGO1	H3K4me2	2VPE (X-ray)	Tyr341 Trp366	Asp352	Val350
	mPYGO	H3K4me3	2YYR (X-ray)	Tyr339 Trp364	Asp350	Val348
	hPYGO2	H3K4me2	4UP0 (X-ray)	Tyr328 Trp353	Asp339	Val337 Asn338 Ala343
	hJARID1A	H3K4me3	3GL6 (X-ray)	Trp1625 Trp1635		
	hPHF2	H3K4me3	3KQI (X-ray)	Tyr7 Tyr14 Trp29		Met20
	hPHF8	H3K4me3	3KV4 (X-ray)	Tyr43 Tyr50 Trp65		Met56 Ser390 Ala392
	hPHF13	H3K4me3	3O7A (X-ray)	Phe241 Trp255		Leu232 Met246
	hPHF20	H3K4me2	5TBN (NMR)	Trp675	Glu662	Val653 Met666
	hMLL1	H3K4me3	3LQJ (X-ray)	Tyr1576 Tyr1581 Trp1594		Ser1583 Met1585
	hMLL5	H3K4me3	4L58 (X-ray)	Trp141	Asp128	Thr119 Met132
	hDIDO3	H3K4me3	4L7X (X-ray)	Tyr270 Trp291	His277	Met282
	scSet3	H3K4me3	5TDW (X-ray)	Trp140	Asp127	Gly116 Ile118 Thr131
	dPPS	H3K4me3	5WLE (X-ray)	Trp912 Trp933	Asp906 His919	Met924
	aAL1	H3K4me3	5Y20 (X-ray)	Tyr195 Trp201 Trp210		Thr186
	aSHL	H3K4me3	5ZNP (X-ray)	Tyr141 Tyr148 Trp163	Asp136	
Tudor	hJMJD2A	H3K4me3	2GFA (X-ray)	Phe932 Trp967 Tyr973	Asp934	Ser936
	hJMJD2A	H4K29me3	2QQS (X-ray)	Phe932 Trp967 Tyr973	Asp934	Ser936
	h53BP1	H4K20me2	2IG0 (X-ray)	Trp1495 Tyr1502 Phe1519 Tyr1523	Asp1521	
	h53BP1	H4K20me2	2LVM (NMR)	Trp1495 Tyr1502 Phe1519 Tyr1523	Asp1521	
	hUHRF1	H3K9me3	2L3R (NMR)	Phe152 Tyr188 Tyr191 Phe237	Asp145	Asn194
	hUHRF1	H3K9me3	3ASK (X-ray)	Phe152 Tyr188 Tyr191	Asp145	Asn194
	hUHRF1	H3K9me3	3DB3 (X-ray)	Phe152 Tyr188 Tyr191	Asp145	Asn194
	hUHRF1	H3K9me3	4GY5 (X-ray)	Phe152 Tyr188 Tyr191	Asp145	Asn194
	hSGF29	H3K4me3	3MEA (X-ray)	Tyr238 Tyr245 Phe264	Asp266	
	hSPINDLIN1	H3K4me3	4H75 (X-ray)	Phe141 Trp151 Tyr170 Tyr177 Tyr179	Asp173	
	hSPINDLIN1	H4K20me3	5Y5W (X-ray)	Phe141 Trp151 Tyr170 Tyr177 Tyr179	Asp173	
	hSPINDLIN4	H3K4me3	4UY4 (X-ray)	Phe127 Trp137 Tyr156 Tyr163 Tyr165	Asp159	

Table S3. Residues forming the binding site for methyl-lysine residues in the histone tail

Domain	Protein	Histone	PDB ID	Residues having side-chains within 6 Å of Nζ of methylated lysine		
				Aromatic	Charged	Others
Tudor	hPHF1	H3K36me3	2M0O (NMR)	Tyr47 Phe65	Asp67	Ser69
	hPHF1	H3K36me3	4HCZ (X-ray)	Trp41 Tyr47 Phe65 Phe71	Asp67	Ser69
	aSHH1	H3K9me3	4IUR (X-ray)	Tyr140 Phe162 Phe165	Asp136 His169 Glu171	Ser133
	hPHF19	H3K36me3	4BD3 (NMR)	Trp50 Tyr56 Phe74 Tyr80	Asp76	Ser78
MBT	hL3MBTL1	H4K20me2	2PQWI (X-ray)	Phe379 Trp382 Tyr386	Asp355	Asn358 Leu361 Cys363 Thr411
	hL3MBTL1	H1.5K27me2	2RHI (X-ray)	Phe379 Trp382 Tyr386	Asp355	Asn358 Leu361 Cys363 Thr411
	hL3MBTL2	H4K20me1	3F70 (X-ray)	Phe570 Trp573 Tyr577	Asp546	Leu552 Cys554
	dSCM	Kme	2R5A (X-ray)	Phe348 Trp351 Phe355	Asp324	Asn327 Leu330 Cys332 Gln380
	hSCML2	Kme	2VYT (X-ray)	Phe206 Trp209 Phe213	Asp182	Asn185 Leu188 Cys190
PWWP	hBRPF1	H3K36me3	2X4W (X-ray)	Tyr1096 Tyr1099 Phe1147	Asp1149	Cys1093 Thr1153
	hZFCW	H3K4me3	2RR4 (NMR)	Trp256 Trp267 Trp303		
	hHDGF2	H3K79me3	3QJ6 (X-ray)	Tyr18 Trp21 Phe44	Glu49	Met15 Thr47
	mZMYND11	H3K36me3	4N4H (X-ray)	Phe291 Trp294 Phe310		Met288 Gln316
	hZCWPW2	H3K4me3	4O62 (X-ray)	Trp30 Trp41 Phe78		
	hDNMT3B	H3K36me3	5CIU (X-ray)	Phe236 Trp239 Trp263	Asp266	Ile233
	mMORC3	H3K4me3	5IX1 (X-ray)	Trp410 Trp419	Glu450 Glu453	
	hMORC3	H3K4me3	5SVX (X-ray)	Trp410 Trp419	Glu450 Glu453	
	hHRP3	H3K36me3	6IIS (X-ray)	Tyr23 Trp26 Phe49	Glu54	Met20 Thr52
WD40	hEED	H1K26me3	3IIY (X-ray)	Phe97 Tyr148 Trp364 Tyr365		Asn194
	hEED	H3K9me3	3IJ0 (X-ray)	Phe97 Tyr148 Trp364 Tyr365		Asn194
	hEED	H3K27me3	3IIW (X-ray)	Phe97 Tyr148 Trp364 Tyr365		Asn194
	hEED	H4K20me3	3IJ1 (X-ray)	Phe97 Tyr148 Trp364 Tyr365		Asn194
	hEED	H3K27me3	3JZG (X-ray)	Phe97 Tyr148 Trp364 Tyr365		Asn194
	hEED	H3K79me3	3JZH (X-ray)	Phe97 Tyr148 Trp364 Tyr365		Asn194
	hEED	H3K4me3	3K26 (X-ray)	Phe97 Tyr148 Trp364 Tyr365		Asn194
	hEED	H3K9me3	3K27 (X-ray)	Phe97 Tyr148 Trp364 Tyr365		Asn194
BAH	mORC1	H4K20me2	4DOW (X-ray)	Tyr63 Trp87 Tyr114 Trp119	Glu93	Val89
	maZMET2	H3K9me2	4FT4 (X-ray)	Tyr203 Trp224 Phe226	Asp230	
	aSHL	H3K27me3	5ZNR (X-ray)	Tyr41 Trp63 Tyr65	Glu69 His88	
Ankyrin	hGLP	H3K9me2	3B95 (X-ray)	Trp874 Trp879 Tyr883 Trp912	Glu882	Ile878
ADD	hATRX	H3K9me3	2LBM (NMR)	Tyr203	Asp207 Glu225	Ile209 Gln219 Ala224
	hATRX	H3K9me3	3QL9 (X-ray)	Tyr203	Asp207 Glu225	Ser206 Ile209 Gln219 Ala224

h : *Homo sapiens*,
m : *Mus musculus*,
d : *Drosophila melanogaster*,
sp : *Schizosaccharomyces pombe*,
sc : *Saccharomyces cerevisiae*,
a : *Arabidopsis thaliana*,
ma : *Zea mays*

Table S4. Residues of Eaf3 CD in which the side-chains are within 6 Å of Nζ of the H3K36me2 analog bound to Eaf3 CD

Residue	Atom	Distance (Å)
18 HIS	Cγ	5.525
18 HIS	Nδ1	5.432
18 HIS	Cδ2	5.091
18 HIS	Cε1	4.888
18 HIS	Nε2	4.655
23 TYR	Cβ	4.789
23 TYR	Cγ	4.128
23 TYR	Cδ1	4.860
23 TYR	Cδ2	3.365
23 TYR	Cε1	4.952
23 TYR	Cε2	3.474
23 TYR	Cζ	4.341
23 TYR	Oζ	5.061
81 TYR	Cβ	4.894
81 TYR	Cγ	4.539
81 TYR	Cδ1	4.336
81 TYR	Cδ2	5.023
81 TYR	Cε1	4.615
81 TYR	Cε2	5.283
81 TYR	Cζ	5.112
81 TYR	Oζ	5.906
84 TRP	Cβ	4.914
84 TRP	Cγ	4.420
84 TRP	Cδ1	4.201
84 TRP	Cδ2	4.489
84 TRP	Nε1	3.974
84 TRP	Cε2	4.135
84 TRP	Cε3	5.211
84 TRP	Cζ2	4.537
84 TRP	Cζ3	5.617
84 TRP	Cη2	5.317
88 TRP	Cβ	4.850
88 TRP	Cγ	4.287
88 TRP	Cδ1	4.495
88 TRP	Cδ2	4.081
88 TRP	Nε1	4.444
88 TRP	Cε2	4.164
88 TRP	Cε3	4.410
88 TRP	Cζ2	4.504
88 TRP	Cζ3	4.749
88 TRP	Cη2	4.792
